# PSGL-1 restricts HIV-1 infectivity by blocking virus particle attachment to target cells

**DOI:** 10.1073/pnas.1916054117

**Published:** 2020-04-09

**Authors:** Yajing Fu, Sijia He, Abdul A. Waheed, Deemah Dabbagh, Zheng Zhou, Benjamin Trinité, Zhao Wang, Jieshi Yu, Dan Wang, Feng Li, David N. Levy, Hong Shang, Eric O. Freed, Yuntao Wu

**Affiliations:** ^a^Key Laboratory of AIDS Immunology of National Health Commission, Department of Laboratory Medicine, The First Affiliated Hospital, China Medical University, Liaoning, 110001 Shenyang, China;; ^b^National Clinical Research Center for Laboratory Medicine, The First Affiliated Hospital, China Medical University, Liaoning, 110001 Shenyang, China;; ^c^National Center for Biodefense and Infectious Diseases, School of Systems Biology, George Mason University, Manassas, VA 20110;; ^d^Virus–Cell Interaction Section, HIV Dynamics and Replication Program, Center for Cancer Research, National Cancer Institute at Frederick, Frederick, MD 21702;; ^e^Department of Basic Science, New York University College of Dentistry, New York, NY 10010;; ^f^Department of Biology and Microbiology, South Dakota State University, Brookings, SD 57006

**Keywords:** HIV-1, PSGL-1, CD43, Vpu, Nef

## Abstract

P-selectin glycoprotein ligand-1 (PSGL-1) is a dimeric, mucin-like, 120-kDa glycoprotein that binds to P-, E-, and L-selectins. PSGL-1 is expressed primarily on the surface of lymphoid and myeloid cells and is up-regulated during inflammation to mediate leukocyte tethering and rolling on the surface of endothelium for migration into inflamed tissues. Although it has been reported that PSGL-1 expression inhibits HIV-1 replication, the mechanism of PSGL-1–mediated anti-HIV activity remains to be elucidated. Here we report that PSGL-1 in virions blocks the infectivity of HIV-1 particles by preventing the binding of particles to target cells. This inhibitory activity is independent of the viral glycoprotein present on the virus particle; the binding of particles bearing the HIV-1 envelope glycoprotein or vesicular stomatitis virus G glycoprotein or even lacking a viral glycoprotein is impaired by PSGL-1. Mapping studies show that the extracellular N-terminal domain of PSGL-1 is necessary for its anti–HIV-1 activity, and that the PSGL-1 cytoplasmic tail contributes to inhibition. In addition, we demonstrate that the PSGL-1–related monomeric E-selectin–binding glycoprotein CD43 also effectively blocks HIV-1 infectivity. HIV-1 infection, or expression of either Vpu or Nef, down-regulates PSGL-1 from the cell surface; expression of Vpu appears to be primarily responsible for enabling the virus to partially escape PSGL-1–mediated restriction. Finally, we show that PSGL-1 inhibits the infectivity of other viruses, such as murine leukemia virus and influenza A virus. These findings demonstrate that PSGL-1 is a broad-spectrum antiviral host factor with a unique mechanism of action.

## Significance

PSGL-1 and CD43 are surface glycoproteins expressed on blood CD4 T cells to bind to selectins for T cell tethering, rolling, and migration into inflamed tissues. The PSGL-1 level is greatly up-regulated during inflammation. Here we found that PSGL-1 and CD43 expression inhibits HIV spreading infection. Mechanistically, PSGL-1 blocks the binding of virus particles to target cells. PSGL-1–mediated suppression of virus infectivity extends to another retrovirus—murine leukemia virus—and to influenza A virus. These results further our understanding of virus–host interactions and help elucidate mechanisms by which cellular host factors regulate viral infection and pathogenesis.

PSGL-1 (P-selectin glycoprotein ligand-1), also known as SELPLG or CD162, is a dimeric, mucin-like, 120-kDa glycoprotein that binds to P-, E-, and L-selectins (1–3). PSGL-1 is primarily expressed on the surface of lymphoid and myeloid cells (1, 4, 5) and is up-regulated during inflammation to mediate leukocyte tethering and rolling on the surface of endothelium, promoting cell migration into inflamed tissues (6–9). PSGL-1 also serves as a surface receptor for enterovirus 71 (EV71) infection of leukocytes (10). In a mouse model of chronic viral infection, PSGL-1 has been reported to regulate T cell checkpoints (11) and has been shown to be an IFN-γ–regulated factor involved in Th1-mediated antiviral activity (8, 12). During T cell differentiation, culturing T cells in the Th1 cytokine IFN-γ and IL-12 promoted PSGL-1 expression preferentially in the IFN-γ–producing T cell population (8), suggesting that PSGL-1 could be an IFN-γ–regulated factor involved in Th1-mediated antiviral activity.

PSGL-1 has been reported to cocluster with HIV-1 Gag at sites of assembly in the T cell uropod in Gag-expressing cells (13). In addition, PSGL-1 was recently identified, through proteomic profiling of HIV-1–infected T cells, as an IFN-γ–induced restriction factor that blocks HIV-1 reverse transcription early postentry and inactivates progeny virion infectivity during viral assembly (12). The anti–HIV-1 activity of PSGL-1 also has been shown to be antagonized by the HIV-1 accessory protein Vpu (12); however, Vpu-mediated PSGL-1 down-regulation occurs only after reverse transcription and Vpu expression. Therefore, how HIV-1 overcomes PSGL-1 restriction at early time points during DNA synthesis is not clear. Furthermore, the mechanism of PSGL-1 inactivation of virion infectivity remains to be elucidated.

In this study, we investigated the molecular details of PSGL-1 restriction of HIV-1 infection of human CD4 T cells. We demonstrate that in a single cycle of HIV-1 infection, ectopic expression of PSGL-1 in target cells did not inhibit HIV infection. However, the presence of PSGL-1 in virus-producing cells inhibited the processing and incorporation of the HIV-1 envelope (Env) glycoprotein, disrupting the attachment of progeny virion to target cells. In the context of VSV-G pseudotyping of HIV-1 virions, particle binding and infectivity were blocked by PSGL-1 without reducing VSV-G incorporation into virions.

These results suggest that the presence of PSGL-1 in virions likely blocks viral infectivity through a structural hindrance of particle binding to target cells. Functional mapping of PSGL-1 demonstrated that the extracellular N-terminal domain of PSGL-1, but not PSGL-1 dimerization, is required for its anti–HIV-1 activity. In addition, we found that a monomeric E-selectin binding glycoprotein CD43 also effectively blocks HIV infectivity with a potency similar to that of PSGL-1, suggesting a shared property of selectin ligands in blocking viral infectivity. Furthermore, we demonstrate that PSGL-1 is a broad-spectrum antiviral host factor, inhibiting the infectivity of multiple viruses.

## Results

### PSGL-1 Is Expressed on Primary CD4 T Cells and Elicits Anti-HIV Activity in HeLa JC.53 Cells.

**Fig. 1.**
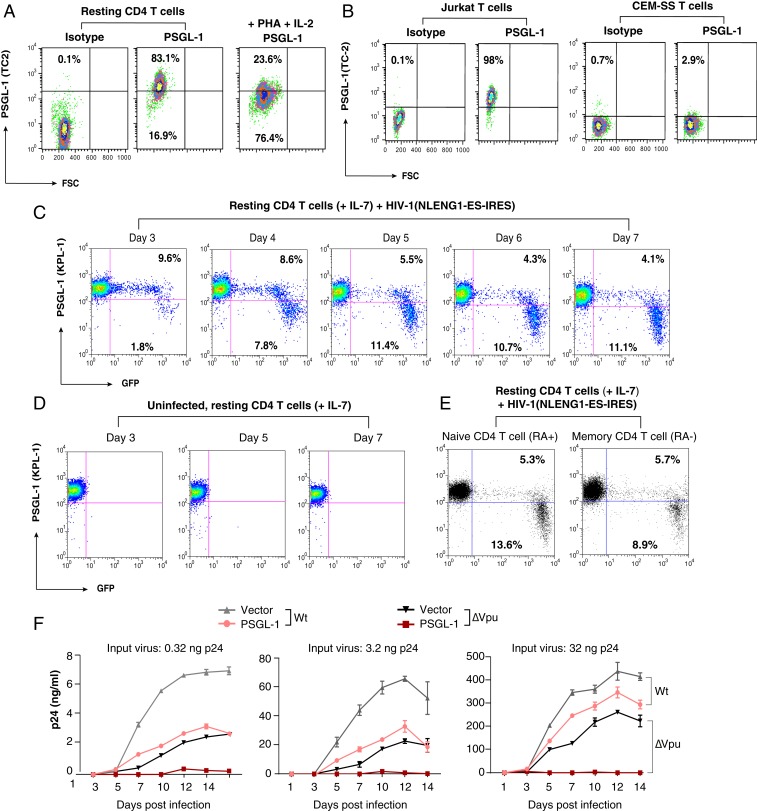
PSGL-1 is expressed on human CD4 T cells and exerts an anti-HIV activity. (*A*) Peripheral blood resting CD4 T cells were purified by negative selection, activated with PHA+IL-2, or left unstimulated. Cell surface PSGL-1 expression was analyzed by flow cytometry. (*B*) Jurkat and CEM-SS cells were similarly stained for surface PSGL-1. (*C*–*E*) Down-regulation of PSGL-1 by HIV-1 in primary CD4 T cells. Primary resting CD4 T cells were infected with NLENG1-ES-IRES, a GFP reporter virus. Following infection, cells were washed and cultured in complete medium plus IL-7 (2 ng/mL) to permit low-level viral replication. Surface PSGL-1 expression was analyzed at the indicated days. (*C*) Percentages of the GFP^+^ or GFP^−^ cells with low or high PSGL-1 staining in each panel. PSGL-1 down-regulation was observed only in the HIV-infected (GFP^+^) cell population. (*D*) For controls, uninfected cells were similarly cultured in IL-7, and surface PSGL-1 expression was analyzed at the indicated days. Culturing of resting CD4 T cells in IL-7 did not lead to PSGL-1 down-regulation. (*E*) Cells were also stained for surface PSGL-1 expression on CD45RA^+^ (naïve) and CD45RA^−^ (memory) CD4 T cells on day 7 and analyzed by flow cytometry. (*F*) HeLa JC.53 cells were stably transfected with PSGL-1 or empty vector DNA and drug-selected to obtain stably transfected cells. These cells were then infected with three different inputs of HIV-1(NL4-3)WT or HIV-1(∆Vpu). Viral replication was quantified by p24 release.

To study the mechanisms of PSGL-1–mediated restriction of HIV-1 replication, we first quantified PSGL-1 expression on both primary blood CD4 T cells and transformed T cell lines. We observed that human blood resting CD4 T cells express high levels of PSGL-1 and that T cell activation (IL-2 plus PHA) down-regulates PSGL-1 (Fig. 1*A*), while transformed CD4 T cell lines express low (e.g., Jurkat) to undetectable (e.g., CEM-SS) levels of PSGL-1 (Fig. 1*B*). Transformed, non-T cell lines, such as HeLa and 293T, do not express detectable levels of PSGL-1 (*SI Appendix*, Fig. S1). Nevertheless, resting T cells can be latently infected and support low levels of HIV replication in the presence of cytokines such as IL-7 (14, 15). It is possible that HIV infection of primary resting CD4 T cells may down-regulate PSGL-1, as seen in transformed T cell lines (12, 16) (*SI Appendix*, Fig. S2). We followed HIV-1 infection of primary resting CD4 T cells and found that HIV-1 infection of IL-7–treated blood resting CD4 T cells down-regulates PSGL-1 exclusively in the HIV-1–positive cell population (Fig. 1 *C* and *D*). HIV-1–mediated down-regulation of PSGL-1 occurred both in the memory and naïve T cell subsets (Fig. 1*E*).

Vpu has been identified as a viral factor that mediates intracellular PSGL-1 degradation (12). However, both Vpu and Nef are known broad-spectrum modulators of cell-surface receptors (16–18). Thus, we also examined the role of Nef in HIV-mediated PSGL-1 down-regulation and observed a dose-dependent down-modulation of surface PSGL-1 levels by both Vpu and Nef, consistent with previous reports (16, 19) (*SI Appendix*, Fig. S3*A*). However, when the levels of intracellular PSGL-1 were examined (*SI Appendix*, Fig. S3*B*), Vpu, but not Nef, was found to cause a decrease in total intracellular PSGL-1. Interestingly, Nef induced an intracellular accumulation of the 70- to 80-kDa species of PSGL-1 (PSGL-1-70) (*SI Appendix*, Fig. S3*B*).

To confirm the anti-HIV activity of PSGL-1 (12), we established PSGL-1–stably transfected HeLa JC.53 cells (HeLaJC53-PSGL-1) (Fig. 1*F*). WT HIV-1 and HIV∆Vpu derivatives were produced in HEK293T cells in the absence of PSGL-1 and then used to infect HeLaJC53-PSGL-1 or control HeLaJC53-empty vector–transfected cells, using p24-normalized inocula. At all three HIV-1 inputs, PSGL-1 inhibited spreading HIV infection over a 14-d time course (Fig. 1*F*). In addition, PSGL-1 displayed a stronger inhibition of HIV∆Vpu than of WT; even at the highest HIV-1 input used (32 ng p24), no spreading viral replication was detected from HIV∆Vpu in HeLaJC53-PSGL-1 cells, whereas WT viral replication was more modestly inhibited (Fig. 1*F*), consistent with previous studies from transient transfection of PSGL-1 into virus-producing cells (12). These results confirm that PSGL-1 restricts the spread of HIV infection, and that Vpu can partially antagonize this restriction.

Given the observed differences between Vpu and Nef in down-regulating PSGL-1 (*SI Appendix*, Fig. S3*A*), we further compared the relative activity of Vpu and Nef in antagonizing PSGL-1. We found that although both Vpu and Nef can down-regulate PSGL-1 from the cell surface, Vpu plays the predominant role in antagonizing PSGL-1; the 50% inhibitory dose (IC_50_) of PSGL-1 for WT HIV-1 (∼2.7 ng) is approximately 10-fold higher than that for HIV∆Vpu (∼0.29 ng). Thus, deletion of Vpu led to a heightened sensitivity to PSGL-1 restriction. In contrast, there was only a slight difference in PSGL-1 IC_50_ between WT and ∆Nef HIV-1 (*SI Appendix*, Fig. S4).

### PSGL-1 Does Not Block Viral Release but Inactivates Virion Infectivity.

**Fig. 2.**
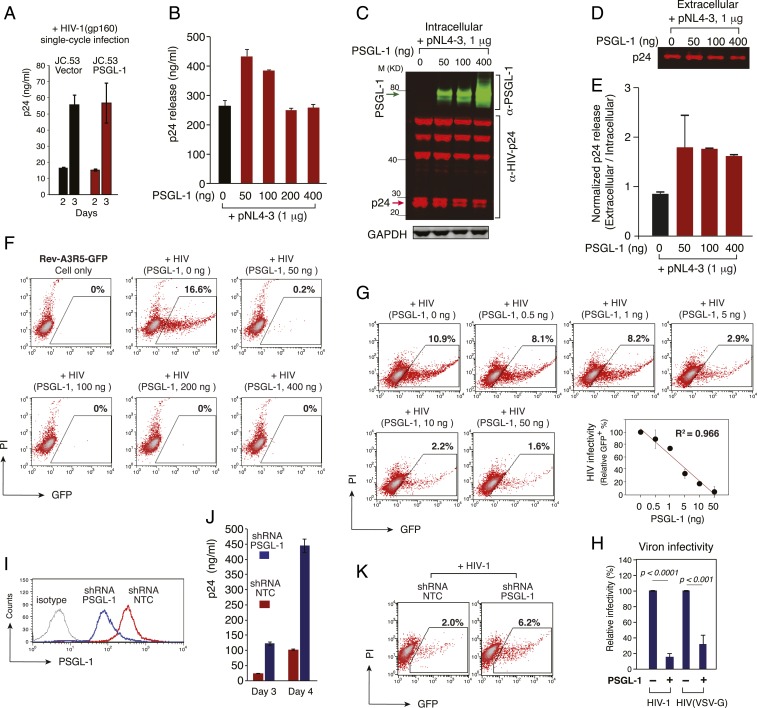
PSGL-1 does not block viral release but inactivates virion infectivity. (*A*) HeLa JC.53 cells were stably transfected with PSGL-1 or empty vector DNA and drug-selected to obtain stably PSGL-1–expressing cells. Cells were then infected with the single-round vector HIV-1 (gp160) for single-round infection. Viral replication was quantified by p24 release. The black and red bars represent empty vector and PSGL-1 vector-transfected cells, respectively. (*B*–*E*) HEK293T cells were cotransfected with HIV(NL4-3) DNA (1 μg) plus different amounts of PSGL-1 expression vector. Viral p24 release was quantified at 48 h (*B*). Cells were also lysed and analyzed by Western blot for intracellular PSGL-1 and HIV-1 proteins (*C*). Extracellular virion p24 was also analyzed by Western blot (*D*), and the relative ratio of extracellular and intracellular p24 was plotted (*E*). The black and red bars represent empty vector and PSGL-1 vector cotransfected cells, respectively. (*F* and *G*) Virions released from HEK293T cells cotransfected with HIV(NL4-3) DNA (1 μg) plus PSGL-1 DNA (0.5 to 400 ng) were harvested at 48 h and normalized for p24, and viral infectivity was quantified by infecting the T cell line-derived Rev-A3R5-GFP indicator cell line. HIV-1 replication was quantified by GFP expression. Shown are the percentages of GFP^+^ cells at 48 h postinfection. (*G*) The PSGL-1 dose-dependent inhibition curve was plotted using results from three independent experiments. (*H*) PSGL-1 blocks the infectivity of VSV-G-pseudotyped HIV-1. Virus particles produced in the presence of PSGL-1 or the empty vector were assayed for infectivity using TZM-bl cells. (*I*–*K*) shRNA knockdown of PSGL-1 enhances virion infectivity. Jurkat cells were transduced with a lentiviral vector expressing shRNA against PSGL-1 (shRNA PSGL-1) or a nontarget sequence (shRNA NTC). PSGL-1 surface expression was quantified (*I*) at 12 d posttransduction and puromycin selection. Cells were also electroporated with HIV-1(NL4-3) DNA, and viral replication in knockdown Jurkat cells was quantified by measuring p24 in the supernatant (*J*). To quantify HIV infectivity, virions were harvested at 48 h postelectroporation and used to infect Rev-A3R5-GFP cells, using an equal amount of p24 for infection (*K*).

PSGL-1 has been suggested to block viral reverse transcription early postinfection and to inactivate virion infectivity late in the replication cycle (12). Thus, we examined the role of PSGL-1 in a single HIV-1 replication cycle. We infected PSGL-1–stably transfected HeLa JC.53 cells (HeLaJC53-PSGL-1) and control cells (HeLaJC53-vector) with an HIV-1 Env-pseudotyped, single-cycle virus, HIV(gp160) (20) (Fig. 2*A*). We observed no inhibition of HIV-1 infection in a single cycle, as judged by the release of virion-associated p24 from the infected cells (Fig. 2*A*). Thus, in our system, PSGL-1 did not block any step in the viral replication cycle up to and including virion assembly and release.

To confirm this result, we cotransfected HEK293T cells with HIV(NL4-3) proviral DNA (1 μg) plus PSGL-1 expression vector at varying inputs from 50 to 400 ng (Fig. 2*B*). We observed small effects of PSGL-1 expression on HIV-1 virion release, from slight enhancement at low doses (<100 ng) to no effect at high doses (200 and 400 ng). When normalized to the levels of intracellular Gag, PSGL-1 did not inhibit virion release at any dose tested (Fig. 2 *C*–*E*). Next, we quantified the infectivity of the released virions on target CD4 T cells, using a highly stringent Rev-dependent indicator cell line, Rev-A3R5-GFP, that does not respond to noninfectious HIV stimuli as do LTR-based reporter cell lines (21–25). As shown in Fig. 2*F*, we observed that PSGL-1 expression in the virus-producer cell almost completely abolished the infectivity of released virions at PSGL-1 vector doses >50 ng (Fig. 2*F*). PSGL-1 partially restricted HIV-1 infectivity at inputs as low as 0.5 ng (PSGL-1:HIV-1 DNA ratio, 1:2,000), and there was a dose-dependent inactivation of HIV-1 at PSGL-1 vector doses of 0.5 to 50 ng (Fig. 2*G*). The potent inactivation of HIV-1 infectivity was confirmed by quantifying HIV infectivity in the HeLa cell-derived TZM-bl cell line (*SI Appendix*, Fig. S5), and by following HIV-1 spreading infection in A3R5.7 CD4 T cells (*SI Appendix*, Fig. S6). Interestingly, we also observed that the infectivity of HIV-1 virions bearing VSV-G was significantly inhibited by PSGL-1 expression in the virus-producer cells (Fig. 2*H* and *SI Appendix*, Fig. S7). Together, these results demonstrate that the presence of PSGL-1 in producer cells inactivates the infectivity of released virions. To corroborate these results, we also produced virions from a human CD4 T cell, CEM-SS. Introduction of PSGL-1 into CEM-SS also inactivated the infectivity of progeny virions released, consistent with the results observed in HEK293T producer cells (*SI Appendix*, Fig. S8).

To further examine the role of endogenous PSGL-1 in virus replication, we performed lentiviral vector-mediated stable shRNA knockdown of PSGL-1 in Jurkat T cells, which express low levels of PSGL-1 (Fig. 1*B*). Following selection, we established a stable pool of Jurkat cells with reduced expression of surface PSGL-1 (shRNA PSGL-1) (Fig. 2*I*). The PSGL-1 knockdown cells, along with the shRNA nontarget control cells (shRNA NTC), were used as the virion producer cells to test the effects of PSGL-1 knockdown on progeny virion infectivity. Cells were directly transfected with HIV-1 DNA by electroporation to produce virions. Following DNA delivery, viral replication in the PSGL-1 knockdown cells was evaluated by measuring extracellular p24. We observed a fourfold to fivefold enhancement of HIV-1 replication in the knockdown cells at days 3 and 4 (Fig. 2*J*).

To confirm that this enhancement was mediated by increased virion infectivity, viral particles were harvested at 48 h, and p24-normalized released virions were used to infect Rev-A3R5-GFP indicator cells. As shown in Fig. 2*K*, we observed enhanced infectivity for virions released from the PSGL-1 knockdown cells. These results are in agreement with the PSGL-1 coexpression experiments (Fig. 2*F*), demonstrating that the presence of PSGL-1 in virus-producer cells, even at low levels such as those present endogenously in Jurkat cells, can inhibit virion infectivity. The levels of PSGL-1 on Jurkat cells are comparable with those on activated primary blood CD4 T cells (Fig. 1 *A* and *B*). To corroborate the PSGL-1 knockdown results observed in Jurkat cells, we also performed similar lentiviral vector-mediated shRNA knockdown of PSGL-1 in preactivated primary CD4 T cells and observed a similar enhancement of HIV-1 replication (*SI Appendix*, Fig. S9).

### PSGL-1 Blocks Virion Attachment to Target Cells.

**Fig. 3.**
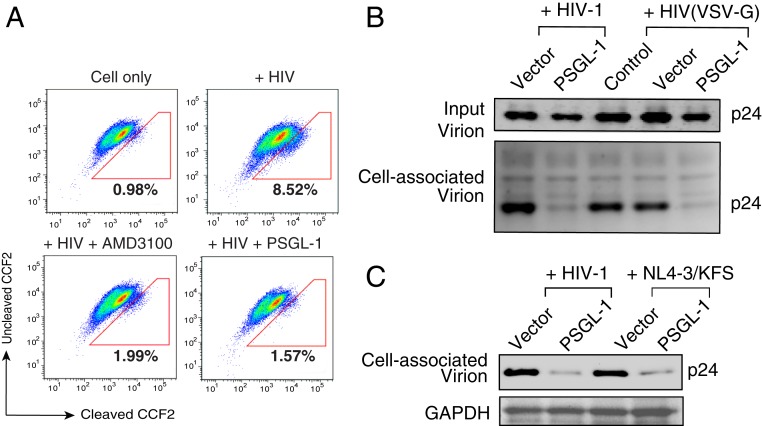
PSGL-1 blocks virion attachment to target cells. (*A*) Virions produced from HEK293T cells cotransfected with PSGL-1 expression vector plus HIV-1(NL4-3) were used for an entry assay. HIV-1 virions similarly produced in the presence of an empty vector served as controls. Equal p24 was used for the assay. The entry inhibitor AMD3100 was also used as a control to block virus entry. The percentages of cells with cleaved CCF2 are shown. (*B*) Virions produced in the presence of PSGL-1 or the empty vector were assayed for attachment to target HeLa JC.53 cells at 4 °C for 2 h. Cells were washed and then analyzed by Western blot for bound p24. (*C*) PSGL-1 blocks Env(−) HIV-1 particle attachment to target cells. WT HIV-1 or Env(−) HIV-1 [(NL4-3)/KFS] virions were produced in the presence of PSGL-1 or the empty vector, and viral particles were assayed for attachment to target HeLa JC.53 cells.

To examine whether PSGL-1 expression in the producer cells and its incorporation into virus particles (*SI Appendix*, Fig. S10) inhibit virus entry, we performed a beta-lactamase–based virus entry assay (26) (Fig. 3*A*). The HIV-1 entry inhibitor AMD3100 served as a positive control for entry inhibition. The results indicated that PSGL-1-imprinted virus particles were severely impaired in their ability to enter target cells (Fig. 3*A*). We next performed a virion attachment assay and observed that virions from PSGL-1–expressing cells were impaired in their ability to attach to susceptible target cells (Fig. 3*B*). Because PSGL-1 expression in the virus-producer cells also inhibits the infectivity of VSV-G–pseudotyped HIV-1 (Fig. 2*H* and *SI Appendix*, Fig. S7), we investigated the effect of PSGL-1 on HIV-1 Env and VSV-G incorporation into virion particles. Whereas we observed that expression of PSGL-1 in the virus-producer cells reduced levels of HIV-1 Env on virions, the levels of the VSV-G on virions were not reduced by PSGL-1 expression (*SI Appendix*, Fig. S11). We also performed a virion attachment assay and observed that PSGL-1–imprinted virions bearing either VSV-G or HIV-1 Env were impaired in their ability to attach to target cells (Fig. 3*B*). These results suggest that the presence of PSGL-1 on virus particles may structurally hinder viral envelope interaction with target cells.

We next tested whether the PSGL-1 inhibition of viral attachment to cells is dependent on the presence of viral envelope proteins. HIV-1 particles devoid of any viral envelope glycoproteins were assembled by transfection of the Env(−) pNL4-3/KFS molecular clone (27) in the presence or absence of the PSGL-1 vector. Virus particles were then used to perform the attachment assay. As shown in Fig. 3*C*, we observed that PSGL-1–imprinted, Env-negative particles were also impaired in their ability to attach to target cells. These results demonstrate that PSGL-1 can inhibit virion attachment to cells in an envelope glycoprotein-independent manner.

### The Extracellular N-Terminal Domain of PSGL-1 Is Required for Blocking HIV-1 Infectivity.

**Fig. 4.**
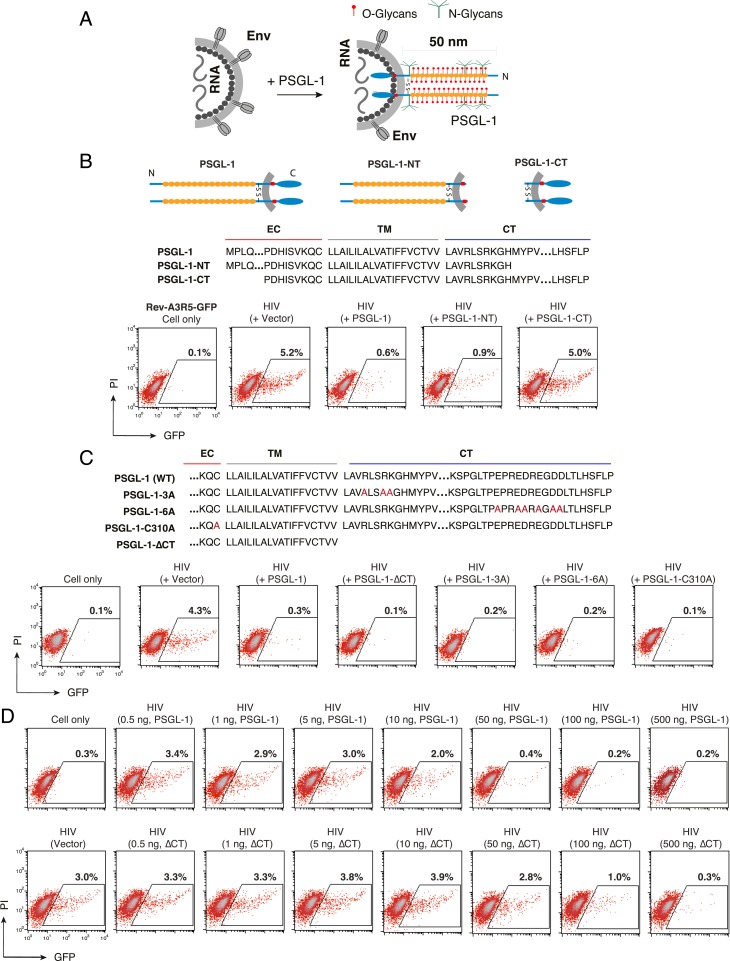
The extracellular N-terminal domain of PSGL-1 is required to block HIV-1 infectivity. (*A*) Hypothetical model by which virion incorporation of PSGL-1 restricts virion infectivity. Based on this model, incorporation of the heavily glycosylated and elongated PSGL-1 on viral particles may interfere with virion binding to target cells. (*B*) PSGL-1 domains involved in blocking HIV-1 infectivity. HEK293T cells were cotransfected with HIV(NL4-3) DNA (1 μg) plus vectors expressing PSGL-1 or PSGL-1 truncation mutants PSGL-1-NT or PSGL-1-CT (500 ng). Virions were harvested at 48 h posttransfection and normalized for p24, and viral infectivity was quantified by infecting Rev-A3R5-GFP indicator cells. HIV-1 replication was quantified by GFP expression. (*C*) PSGL-1 intracellular domain mutants PSGL-1-∆CT, PSGL-1-3A, and PSGL-1-6A and the dimerization mutant PSGL-1-C310A were similarly tested. EC, extracellular domain; TM, transmembrane domain; CT, cytoplasmic tail. (*D*) The PSGL-1-∆CT mutant displays reduced antiviral activity relative to WT PSGL-1. HEK293T cells were cotransfected with HIV(NL4-3) DNA (1 μg) plus various amounts of PSGL-1 or PSGL-1-∆CT (0.5 to 500 ng). Virions were harvested at 48 h and normalized for p24, and their infectivity was measured in Rev-A3R5-GFP indicator cells.

Structurally, PSGL-1 has a relatively rigid and elongated extracellular domain that extends nearly 60 nm from the cell surface (28, 29). To test the hypothesis that the presence of PSGL-1 on virus particles may sterically hinder the binding of particles to target cells (Fig. 4*A*), we performed deletion mutagenesis of PSGL-1 domains. The extracellular N-terminal or intracellular C-terminal domains of PSGL-1 were deleted to generate PSGL-1-CT or PSGL-1-NT (Fig. 4*B*). Vectors expressing PSGL-1 (pRetroPSGL-1), PSGL-1-CT (pRetroPSGL-1-CT), or PSGL-1-NT (pRetroPSGL-1-NT) were cotransfected with HIV(NL4-3) DNA into HEK293T cells to produce viral particles (*SI Appendix*, Fig. S12). Cellular expression and virion incorporation of PSGL-1 mutants were confirmed by surface staining and Western blot analysis (*SI Appendix*, Fig. S12). Virion infectivity was subsequently quantified in Rev-A3R5-GFP cells. As shown in Fig. 4*B*, deletion of most of the intracellular C-terminal domain had a minimal effect on PSGL-1’s ability to restrict HIV-1, whereas truncation of the extracellular N-terminal domain largely abolished the restrictive phenotype. These results demonstrate that the extracellular N terminus of PSGL-1 is required for its anti–HIV-1 activity.

To confirm and extend these results, we tested an additional PSGL-1 N-terminal deletion mutant, pPSGL-1∆DR, in which the decameric repeats (DRs) were removed (30); a large structural part of PSGL-1’s extracellular domain consists of 14 to 16 decamers, which are characterized by repeated stretches of 10 amino acids with numerous *O*-glycosylated threonines (30%) and prolines (10%) (30, 31). DRs play a pivotal role in elongating and strengthening the protein backbone to extend the N-terminal selectin-binding sites far away from the cell surface to support and stabilize leukocyte rolling on L- or P-selectin (30). Indeed, when the DR was deleted from the N terminus, the anti-HIV activity of PSGL-1 was also abolished (*SI Appendix*, Fig. S13).

We further tested a panel of previously studied PSGL-1 mutants (13) (Fig. 4*C* and *SI Appendix*, Fig. S12). PSGL-1-∆CT has the cytoplasmic tail completely removed, whereas PSGL-1-3A and PSGL-1-6A have multiple amino acid changes in the cytoplasmic tail; PSGL-1-3A has the three juxtamembrane basic residues changed to alanine, and PSGL-1-6A has the six acidic residues near the C terminus replaced with alanine (Fig. 4*C*). An additional mutant, PSGL-1-C310A, abolishes PSGL-1 dimerization (32). Given that the cytoplasmic tail mutations may decrease virion incorporation of PSGL-1 (13), we used a high amount of the mutant PSGL-1 DNA (500 ng) to cotransfect with HIV-1 DNA (1 μg) to ensure that even with a lower level of virion incorporation of PSGL-1, it would still be sufficient to confer a restrictive phenotype. Using this high PSGL-1 dose, we were able to demonstrate that all of the PSGL-1 C-terminal mutants, along with the dimerization mutant C310A, maintained their capacity to restrict HIV-1 (Fig. 4*C*), confirming that the N-terminal domain of PSGL-1 is primarily responsible for its anti-HIV activity, and that PSGL-1 dimerization is not required.

Although deletion of the C-terminal domain of PSGL-1 did not abolish its anti-HIV activity (Fig. 4*C*), the complete removal of the C terminus of PSGL-1 has been reported to reduce PSGL-1 coclustering with Gag (13) and thus may affect its ability to be incorporated into HIV-1 particles and inhibit viral infection. To investigate this possibility, we performed a side-by-side dose–response comparison of the ability of WT PSGL-1 and PSGL-1-∆CT to inactivate virion infectivity. As shown in Fig. 4*D*, the complete removal of the C-terminal cytoplasmic tail of PSGL-1 (PSGL-1-∆CT) led to a 10-fold reduction in PSGL-1’s anti-HIV activity. These results demonstrate that although the C terminus is not absolutely required, it can affect PSGL-1’s anti-HIV capacity, likely by inducing coclustering with Gag to promote virion packaging.

### PSGL-1 Is a Broad-Spectrum Antiviral Host Factor.

Given that the PSGL-1 intracellular domain deletion mutant PSGL-1-∆CT effectively blocks HIV-1 infectivity in the absence of coclustering with HIV-1 Gag (13), it is possible that PSGL-1 does not rely on specific interactions with an HIV-1 protein to restrict particle infectivity. Indeed, PSGL-1 inhibition of viral attachment to cells is independent of the presence of viral envelope protein (Fig. 3*C*). The mere presence of the long extracellular domain of PSGL-1 on the surface of virus particles may be sufficient to interfere with particle binding to target cells (Fig. 4*A*). Thus, PSGL-1 may possess a broad-spectrum antiviral activity that extends beyond HIV-1.

**Fig. 5.**
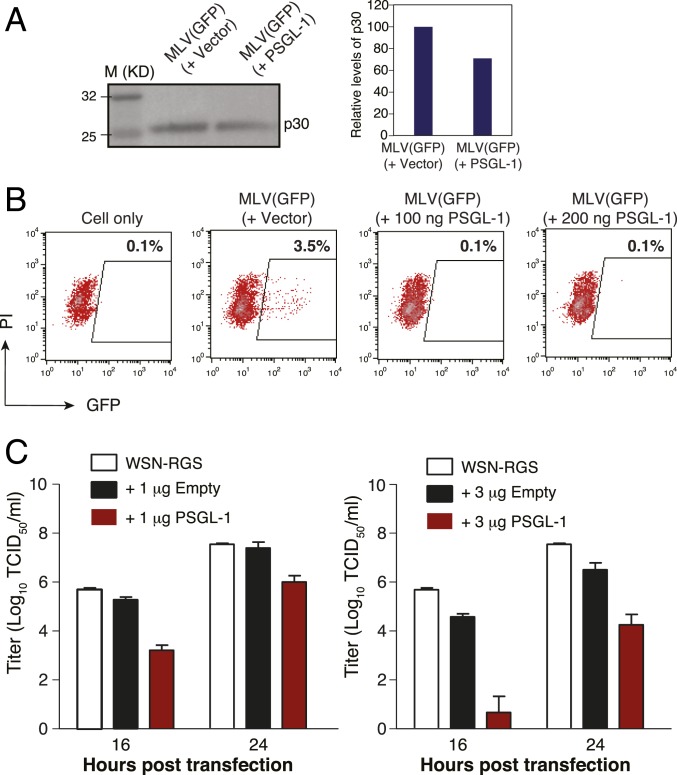
PSGL-1 restricts MLV and influenza A virus infectivity. (*A*) HEK293T cells were cotransfected with an MLV helper vector, pSV-ψ-MLV-env^−^, pRetroQ-AcGFP-N1, pHCMV-G, plus a PSGL-1 expression vector or an empty control vector. Virions were harvested and quantified by Western blot analysis using an anti-MLV p30 (CA) protein antibody. (*B*) MLV virions were harvested, and viral infectivity was quantified by infecting HEK293T cells and measuring GFP expression. (*C*) Eight vectors expressing each of the segments of the influenza A/WSN/33 (H1N1) genome were cotransfected with a PSGL-1 expression vector into HEK293T-MDCK cells. Viral particles were harvested at 16 and 24 h after cotransfection, and virion infectivity was quantified by a TCID_50_ assay.

To test this hypothesis, we produced VSV-G–pseudotyped murine leukemia virus (MLV) particles in the presence of PSGL-1. Although PSGL-1 expression did not block MLV particle production (Fig. 5*A*), it did severely inactivate MLV infectivity (Fig. 5*B*).

Finally, we tested the ability of PSGL-1 to disrupt the infectivity of a nonretroviral enveloped virus, influenza A virus. Eight vectors expressing each of the segments of the influenza A/WSN/33 (H1N1) genome were cotransfected with the PSGL-1 vector into HEK293T-MDCK cocultured cells. Viral particles were harvested at 16 and 24 h posttransfection, and virion infectivity was quantified by the TCID_50_ assay. As shown in Fig. 5*C*, at 16 h, cotransfection with 1 μg of PSGL-1 led to a 100-fold reduction in the infectious titer from released virions, whereas with 3 μg of PSGL-1, the viral titer was reduced by >8,000-fold. These results demonstrate that PSGL-1 has a broad antiviral activity.

### PSGL-1–Related Selectin Ligand CD43 also Restricts HIV Infectivity.

Given that PSGL-1 restriction of HIV infectivity does not require its dimerization (Fig. 4*C*), we speculated that monomeric PSGL-1 and related selectin ligands may also inactivate virion infectivity. Therefore, we further tested CD43, a monomeric glycoprotein that, like PSGL-1, binds E-selectin (33, 34). Similar to PSGL-1, CD43 is a mucin-like protein that is heavily glycosylated (35). CD43 is also bulky and protrudes 45 nm from the cell surface (36). The glycosylated form of CD43 is expressed in Th1 T cells and functions as an E-selectin–specific ligand (33); CD43 collaborates with PSGL-1 to mediate E-selectin–dependent T cell migration (37). We cotransfected HEK293T cells with HIV(NL4-3) proviral DNA (1 μg) plus a CD43 expression vector at varying inputs from 0.5 to 400 ng (*SI Appendix*, Fig. S14), then quantified the infectivity of the released virions on Rev-A3R5-GFP cells. We observed that CD43 almost completely abolished the infectivity of released virions at CD43 vector doses >50 ng (*SI Appendix*, Fig. S14*A*). CD43 partially restricted HIV-1 infectivity at inputs as low as 0.5 to 1 ng, and there was a dose-dependent inactivation of HIV-1 at CD43 vector doses of 0.5 to 50 ng. The IC_50_ of CD43 for inhibiting HIV-1 was 2.69 ng, similar to that of PSGL-1 (∼2.7 ng) (*SI Appendix*, Fig. S14*B*). These results suggest that the shared structural properties (extensive glycosylation, 40- to 60-nm protrusion from the surface, and a rigid, rod-like structure) of selectin ligands may pose a broad issue for viruses if incorporated and displayed on virion surfaces.

## Discussion

In this study, we demonstrate that PSGL-1, a mucin-like glycoprotein highly expressed on blood CD4 T cells, restricts HIV-1 virion infectivity primarily by interfering with particle binding to target cells. In contrast to the Vpu-antagonized restriction factor BST2/tetherin, which tethers virus particles to the cell surface (38, 39), PSGL-1 does not inhibit virion release. PSGL-1 is a remarkably potent inhibitor; it almost completely inactivates WT HIV-1 particle infectivity at a vector:proviral DNA ratio of 0.05:1 (Fig. 2*G*). In the context of VSV-G–pseudotyped HIV-1 particles, PSGL-1 expression markedly restricts particle infectivity without reducing VSV-G incorporation. It has been noted that HIV-1 particles bind target cells even in the absence of Env–receptor interactions (40–42). Remarkably, even when HIV-1 particles are devoid of viral glycoproteins, PSGL-1 in the producer cells interferes with particle binding to target cells. We postulate that in addition to direct effects on Env processing and incorporation (*SI Appendix*, Fig. S11 *A* and *B*), the presence of PSGL-1 on the virion surface may sterically disrupt the binding of virions to target cells. PSGL-1 has a highly extended structure, with the extracellular portion projecting nearly 60 nm from the cell surface (28, 29), and the extracellular domain is heavily glycosylated and relatively rigid (43, 44). These intrinsic structural features (Fig. 4*A*) may occlude the interaction of the particle with viral glycoprotein receptors and may also reduce nonspecific binding between the virion and cells.

PSGL-1 expression was strongly inhibitory in our HIV-1 spreading assay, in which much of the viral transmission likely occurs through cell-to-cell contact at virologic synapses. Although the effects of PSGL-1 on HIV-1 cell-to-cell transmission have not yet been studied, given that PSGL-1 is recruited to sites of viral assembly by Gag (13), we speculate that it is likely present and inhibitory at the virologic synapse. Interestingly, it has been shown that overexpression of PSGL-1 in cells also inhibits antibody binding to multiple cell surface receptors (45); the molecular mechanism has been suggested to be steric hindrance of antibody access to surface molecules resulting from the extended structure and heavy *O*-glycosylation of the extracellular domains of PSGL-1 (45).

Given the detrimental effect of PSGL-1 in hindering particle binding to target cells, one would expect viruses to evolve strategies to remove PSGL-1 from the virion surface. HIV-1 appears to use at least two viral accessary proteins, Vpu and Nef, to down-regulate PSGL-1 from the surface (*SI Appendix*, Fig. S3*A*). Nevertheless, Vpu and Nef may antagonize PSGL-1 by different mechanisms. Vpu binds to PSGL-1 and induces its ubiquitination and degradation (12), whereas Nef does not reduce intracellular PSGL-1 levels. Nef may redirect PSGL-1 to intracellular compartments, resulting in the accumulation of the 70- to 80-kDa species (PSGL-1-70) (*SI Appendix*, Fig. S3*B*). Currently, the molecular identity of PSGL-1-70 is not known. PSGL-1-70 may be a PSGL-1 species with different posttranslational modifications (e.g., *N*- or *O*-linked glycosylation) relative to the larger species. The biological role of this PSGL-1-70 species is not currently known. It also remains to be determined whether the 70- to 80-kDa PSGL-1 species is produced in HIV-1–infected primary cells expressing endogenous PSGL-1. Although Nef is able to reduce cell surface levels of PSGL-1, Vpu appears to play the dominant role in counteracting the antiviral effects of this inhibitory factor. In the case of CD43, both Vpu and Nef are able to down-regulate cell surface expression.

In addition to disrupting the binding of HIV-1 virions to target cells, we observed that PSGL-1 expression in the virus-producer cells is also capable of interfering with the infectivity of another retrovirus—MLV—as well as the influenza A virus. We and others have provided evidence that HIV-1 antagonizes PSGL-1 through the activities of Vpu and Nef. Further work is needed to determine whether other retroviruses and nonretroviral enveloped viruses have also acquired defense mechanisms to counteract the roadblock imposed by PSGL-1.

The work reported here provides insights into the ability of the host cell to interfere with infection by HIV-1 and other enveloped viruses, as well as the biological function of lentiviral accessory proteins. Further elucidation of PSGL-1’s interaction with HIV-1 and other viruses may offer therapeutic strategies for targeting viral infections.

## Materials and Methods

### Cells and Viruses.

Peripheral blood buffy coats from HIV-1–negative adults were purchased from the New York Blood Center or received from the NIH Blood Bank. CD4^+^ T cells were isolated by negative selection as described previously (46). HeLaJC.53 cells were kindly provided by David Kabat. PSGL-1-HeLaJC53 was derived from HeLaJC.53 by transfection with 2 μg of pCMV3-PSGL-1 DNA. TZM-bl cells were obtained through the NIH AIDS Reagent Program, Division of AIDS (John C. Kappes and Xiaoyun Wu). The HIV Rev-dependent GFP indicator Rev-A3R5-GFP cells were provided by Virongy. Additional descriptions of cells and cell culture conditions are provided in *SI Appendix*, *Materials and Methods*.

### Plasmids, Vectors, Transfection, and Virion Production and Purification.

The infectious HIV-1 molecular clone pNL4-3, codon-optimized Vpu expression vector (pcDNA-Vphu), Nef expression vector (pNef-ER), and NL4-3 ∆Vpu infectious molecular clone (pNL-U35) were obtained from the NIH AIDS Reagent Program. pCMV3-PSGL-1 and pCMV3-empty vectors were obtained from Sinobiological. pRetroPSGL-1-NT, pRetroPSGL-1-CT, and pRetroPSGL-1 were synthesized and cloned into pMSCVneo vector by GeneScript. pPSGL-1-3A, pPSGL-1-6A, pPSGL-1-C310A, pPSGL-1-∆CT, and pPSGL-1(Wt) were kindly provided by Akira Ono (13, 47). pPSGL-1∆DR was kindly provided by Caroline Spertini and Olivier Spertini (30). pCMV3-CD43 was obtained from Sinobiological. pSV-ψ-MLV-env^−^ was obtained from the NIH AIDS Reagent Program. pNL∆ΨEnv (gp160) and pHCMV-G expressing the HIV-1 Env and the vesicular stomatitis virus G glycoprotein, respectively, were described previously (48). The GFP-expressing retroviral vector pRetroQ-AcGFP1-N1 was obtained from Clontech. pNL4-3∆Nef was described previously (46). The *env*-defective pNL4-3 derivative pNL4-3/KFS was described previously (27). Detailed procedures for HIV-1 particle production were described previously (46). Additional descriptions of viral particles assembly and purification are provided in *SI Appendix*, *Materials and Methods*.

### Viral Attachment Assay.

Virion particles produced in the presence of PSGL-1 or the empty vector were incubated with HelaJC.53 cells (prechilled at 4 °C for 1 h) at 4 °C for 2 h. The cells were then washed extensively (five times) with cold PBS buffer and then lysed with LDS lysis buffer (Invitrogen) for analysis by Western blot.

### Infectivity Assays.

For flow cytometry-based infectivity assay, virus particles were produced in HEK293T cells by cotransfection with pNL4-3, pNL4-3∆Vpu, or pNL4-3∆Nef with pCMV3-PSGL-1, pCMVCD43, or pCMV3-empty vector, or by cotransfection with pNL4-3/KFS, pHCMV-G, and pCMV3-PSGL-1 or pCMV3-empty vector. Viral particles were also produced in CEM-SS cells by electroporation. Rev-A3R5-GFP cells were infected with each of the indicated viruses (0.2 to 0.5 million cells/infection), and GFP expression was quantified by flow cytometry. For MLV virion infectivity, an MLV-GFP reporter virus was assembled by cotransfecting HEK293T with pSV-Ψ-MLV-env^−^, pRetroQ-AcGFP1-N1, pHCMV-G, and pCMV3-PSGL-1 or an empty vector. Viral supernatants were harvested and used to infect HEK293T cells in the presence of Infectin (Virongy). Virion infectivity was quantified by measuring GFP expression by flow cytometry. Additional information on the viral infectivity assays is provided in *SI Appendix*, *Materials and Methods*.

### ShRNA Knockdown of PSGL-1.

Lentiviral vectors carrying shRNAs against PSGL-1 were purchased from Sigma-Aldrich. Virion particles were assembled by cotransfecting HEK293T cells with pHCMV-G, pCMV-ΔR8.2, and lentiviral vectors. Supernatant was collected and used to transduce Jurkat T cells. Cells were selected in puromycin. PSGL-1 knockdown was confirmed by surface staining with an anti–PSGL-1 antibody (KPL-1; BD Pharmingen). Lentiviral vector-mediated ShRNA knockdown of PSGL-1 in primary CD4 T cells was performed as described previously (46). In brief, blood resting CD4 T cells were purified by negative depletion, transiently stimulated with anti-CD3/CD28 beads, and then transduced with the lentiviral vectors carrying shRNAs against PSGL-1. Additional details of shRNA knockdown and analysis are provided in *SI Appendix*, *Materials and Methods*.

### Data Availability.

All data generated or analyzed during this study are included in this article and its extended data file. Reagents are available from Y. W. on request.

## Supplementary Material

Supplementary File
