# Supplementary material for: PSGL-1 restricts HIV-1 infectivity by blocking virus particle attachment to target cells
Source: Proc Natl Acad Sci U S A. 2020 Apr 9;117(17):9537–45. doi: 10.1073/pnas.1916054117 (PMC7196789; doi:10.1073/pnas.1916054117)
Supplement: Supplementary File [file pnas.1916054117.sapp.pdf]

PNAS
www.pnas.org

Supplementary Information for

PSGL-1 restricts HIV-1 infectivity by blocking virus particle attachment to target cells

Yajing Fu, Sijia He, Abdul A. Waheed, Deemah Dabbagh, Zheng Zhou, Benjamin Trinité, Zhao Wang, Jieshi Yu, Dan Wang, Feng Li, David N. Levy, Hong Shang, Eric O. Freed, and Yuntao Wu

Yuntao Wu, Eric O. Freed, and Hong Shang

Email: ywu8@gmu.edu
efreed@mail.nih.gov
hongshang100@hotmail.com

This PDF file includes:

Supplementary text
Figures S1 to S14

Materials and Methods

Cells and viruses. Peripheral blood buffy coats from HIV-1-negative adults were purchased from the New York Blood Center or received from the NIH Blood Bank. CD4+ T cells were isolated by negative selection using the Dynabeads Untouched magnetic separation kit (Invitrogen) or as previously described (1). CD4+ T cells were cultured in RPMI 1640 plus 10% fetal bovine serum (FBS) and 1x penicillin-streptomycin (Invitrogen). Resting CD4 T cells were activated by culturing in PHA (2 μg/ml) plus IL-2 (2 ng/mL) (PepTech). HEK293T cells (ATCC) and HeLaJC.53 cells (kindly provided by Dr. David Kabat) were maintained in Dulbecco-modified Eagle's medium (DMEM) (Invitrogen) containing 10% FBS and 1x penicillin-streptomycin (Invitrogen). PSGL-1-HeLaJC.53 and Empty-HeLaJC53 cells were cultured in DMEM supplemented with 10% FBS and 550 μg/ml hygromycin B (Invitrogen). TZM-bl cells (Dr. John C. Kappes, and Dr. Xiaoyun Wu (2)) were obtained through the NIH AIDS Reagent Program, Division of AIDS, NIAID, NIH. TZM-bl cells were cultured in DMEM containing 10% FBS and 1x penicillin-streptomycin (Invitrogen). Jurkat cells (NIH AIDS Reagent Program) were cultured in RPMI 1640 supplemented with 2 mM L-glutamine, 10% FBS and 1x penicillin-streptomycin (Invitrogen). HIV Rev-dependent GFP indicator Rev-A3R5-GFP cells (Virongy) were cultured in RPMI 1640 plus 10% FBS supplemented with 1 μg/ml G418 (Sigma-Aldrich) and 1 μg/ml puromycin (Sigma-Aldrich). A3R5.7 cells (NIH AIDS Reagent Program) were cultured in RPMI-1640 containing 10% FBS, 1% L-Glutamine, 1x penicillin-streptomycin, and 1 μg/mL G418 (Invitrogen). CEM-SS cells (NIH AIDS Reagent Program) were cultured in RPMI-1640 with 10% FBS. To construct PSGL-1-HeLaJC53 cells, HeLaJC.53 cells were seeded into a 6-well plate and cultured in DMEM with 10% FBS. Cells were transfected with 2 μg pCMV3-PSGL-1 or pCMV3-Empty DNA using Jetprime transfection reagent (Polyplus) as recommended by the manufacturer. Transfected cells were cultured and selected with DMEM containing 10% FBS and 550 μg/ml of hygromycin B (Invitrogen) to generate stably transfected cells.

Plasmids, vectors, transfection, and virion production and purification. The infectious HIV-1 molecular clone pNL4-3, codon-optimized Vpu expression vector (pcDNA-Vphu), Nef expression vector (pNef-ER), and NL4-3 ΔVpu infectious molecular clone (pNL-U35) were obtained from the NIH AIDS Reagent Program. pCMV3-PSGL-1 and pCMV3-Empty vectors were obtained from Sinobiological. pRetroPSGL-1-NT, pRetroPSGL-1-CT, and pRetroPSGL-1 were synthesized and cloned into pMSCVneo vector by GeneScript. pPSGL-1-3A, pPSGL-1-6A, pPSGL-1-C310A, pPSGL-1-ΔCT, and pPSGL-1(Wt) were kindly provided by Dr. Akira Ono (3, 4). pPSGL-1ΔDR was kindly provided by Drs. Caroline Spertini and Olivier Spertini (5). pCMV3-CD43 was obtained from Sinobiological. pSV-ψ-MLV-env- was from NIH AIDS Reagent Program. pNLΔΨEnv (gp160) and pHCMV-G expressing the HIV-1 Env and the vesicular stomatitis virus G glycoprotein, respectively, were described previously (6). The GFP-expressing retroviral vector pRetroQ-AcGFP1-N1 was obtained from Clontech. pNL4-3ΔNef was described previously (1). The *env*-defective pNL4-3 derivative pNL4-3/KFS was described previously (7).

The procedure for transfection of HEK293T cells to produce HIV-1 particles was described previously (1). For GFP reporter MLV particle assembly, pRetroQ-AcGFP1-N1 (0.5 μg), pSV-ψ-MLV-env- (0.375 μg), and pHCMV-G (0.125 μg) were cotransfected with either pCMV3-PSGL-1 or pCMV3-Empty vector (at indicated dosages) in a 6-well plate. Virus supernatants were collected at 48 hours post transfection. For transient transfection of HeLaJC.53 cells, 0.5 million cells were transfected with 2 μg of either pCMV3-Empty or pCMV-PSGL-1 using the transfection reagent Jetprime (Polyplus) as recommended by the manufacturer. Following transfection, cells were cultured for the indicated times until analysis. For the p24 release assay in HEK293T cells, cells were cotransfected with 1 μg of HIV-1 NL4-3 and indicated doses of pCMV3-PSGL-1 or pCMV3-Empty DNA using Lipofectamine 2000 (Invitrogen). Supernatant was collected at 48 hours posttransfection. To purify virions by ultracentrifugation, supernatants harvested from transfected HEK293T cells first filtered through a 0.45um filter, then concentrated by Vivaspin20 concentrator. Concentrated viruses were purified by ultra-speed centrifugation through a gradient of 6-18% OptiPrep solution (Sigma-Aldrich) (40,000 rpm for 2 hours, SW41Ti rotor from Beckman) , followed by a second round of ultracentrifugation to pellet the virus (20,000 rpm for 1.5 hours, SW41Ti).

FACS analysis. For PSGL-1 surface staining, 0.5–1 million cells were stained with anti-PSGL1 antibody (KPL-1) (BD Pharmingen) followed by staining with Alexa Fluor 488-conjugated goat anti-mouse secondary antibody (Invitrogen). For surface staining of infected blood resting CD4+ T cells, HIV-1 infection was done using 125 ng to 320 ng p24 gag equivalents of NLENG1-ES-IRES GPF reporter virus (8, 9) (pseudotyped with HIV-1 NL4-3 envelope) per million cells. Cells were washed and cultured in 10% FBS RPMI with IL-7 (2 ng/mL) (10). On the indicated days, cells were harvested and stained at 4°C for 30 min with AF687 anti-PSGL-1 antibody (KPL-1, BD Pharmingen) and analyzed by flow cytometry. For surface PSGL-1 staining of Jurkat, CEM-SS, and A3R5.7 cells, 0.5 million cells were stained with FITC-conjugated anti-PSGL-1 antibody (Abcam) and analyzed by flow cytometry. For HIV-1-infected Jurkat T-cell surface staining, 0.5 million cells were infected with different volumes of HIV-1 NL4-3. At 3 days post infection, cells were stained with anti-PSGL-1 antibody [KPL-1] (BD Pharmingen), followed by staining with Alexa Fluor 488-conjugated goat anti-mouse secondary antibody (Invitrogen) and flow cytometry analysis. For HEK293T cells, 0.5 million cells were cotransfected with different dosages (1 μg to 4 μg) of HIV NL4-3 Vpu or HIV NL4-3 Nef, and 100 ng of pCMV3-PSGL-1 using Lipofectamine 2000 (Invitrogen). Cells were stained at 48 hours posttransfection with anti-PSGL-1 antibody [KPL-1] (BD Pharmingen), followed by staining with Alexa Fluor 488-conjugated goat anti-mouse secondary antibody (Invitrogen).

Western blots. The following antibodies were from the NIH AIDS Reagent Program: anti-HIV-1 p24 monoclonal antibody (183-H12-5C), anti-HIV Env (16H3) antibody, anti-HIV-1 gp41 monoclonal antibody (2F5), anti-HIV-1 gp41 monoclonal antibody (10E8), and anti-HIV immune globulin (HIVIG). Cells or virus pellets were solubilized in lysis buffer containing 50 mM Tris-HCl (pH 7.4), 150 mM NaCl, 1 mM EDTA, 0.5% Triton X-100, and protease inhibitor cocktail (Roche Life Science, Basel, Switzerland) or LDS lysis buffer (Invitrogen). Proteins were denatured by boiling in sample buffer and subjected to SDS-PAGE, transferred to PVDF or nitrocellulose membrane, and incubated overnight at 4°C with one of the following primary antibodies: anti-PSGL-1 monoclonal antibody (clone KPL-1, BD Pharmingen) (1:1000 dilution); anti-PSGL-1 TC-2 monoclonal antibody (Abcam) (1:1000 dilution); anti-PSGL-1 polyclonal antibody (Abcam) (1:1000 dilution); anti-PSGL-1-C terminal polyclonal antibody (anti-PSGL-1 amino acid 350 to the C-terminus) (Abcam) (1 μg/ml); anti-GAPDH goat polyclonal antibody (Abcam) (1:1000 dilution); anti-CD45RA antibody (BD Biosciences); anti-HIV envelope antibodies (183-H12-5C, 16H3, 2F5), HIVIG, and anti-CD63 polyclonal antibody (System Biosciences) (1:1000 dilution). Membranes were then incubated with HRP-labeled goat anti-mouse IgG (KPL) (1:2500 dilution) or anti-rabbit IgG (Cell Signaling) (1:2000 dilution) for 60 min at room temperature. Chemiluminescence signal was detected by using West Pico or West Femto chemiluminescence reagent (Thermo Fisher Scientific). Images were captured with a CCD camera (FluorChem 9900 Imaging Systems) (Alpha Innotech). Protein bands were also quantified using Imagelab-Chemidoc (Bio-Rad Laboratories, France). On some occasions, western blot was also performed using infrared imaging (Odyssey infrared imager, LI-cor Biosciences) with IRDye goat anti-mouse or rabbit 680 or 800 cw labeled antibodies (Li-cor Biosciences) (1:5000 diluted in blocking buffer) for 1h at 4°C. The blots were washed three times for 15 minutes and scanned with Odyssey Infrared Imager (Li-cor Biosciences). The ratios of gp120/p24 and gp160/p24 were quantified in virions, and the ratio of gp120/gp160 and expression of PSGL-1 were quantified in cell and virus fractions. To quantify virus release efficiency, HEK293T cells were transfected with the indicated plasmids (WT pNL4-3, pNL43ΔVpu, or pNL43ΔNef) in the absence or presence of PSGL-1 expression vector using Lipofectamine 2000 (Invitrogen) or polyethylenimine (PEI) transfection reagent (Sigma-Aldrich). At 30 to 48 hours after the addition of DNA, virus-containing supernatant was harvested for p24 ELISA, or filtered and pelleted in an ultracentrifuge for analysis. The viral release efficiency (VRE) was calculated as the amount of virion-associated Gag as a fraction of total (cell- and virion-associated) Gag quantified from Western blot analysis.

p24 ELISA. HIV-1 p24 released into the cell culture supernatant was detected by an in-house p24 ELISA kit. Briefly, microtiter plates (Sigma-Aldrich) were coated with anti-HIV-1 p24 monoclonal antibody (183-H12-5C) (NIH AIDS Reagent Program). Samples were incubated for 2

hours at 37ºC, followed by washing and incubating with biotinylated anti-HIV immune globulin (HIVIG) (NIH AIDS Reagent Program) for 1 hour at 37ºC. Plates were then washed and incubated with avidin-peroxidase conjugate (R &D Systems) for 1 hour at 37° C, followed by washing and incubating with tetramethylbenzidine (TMB) substrate. Plates were kinetically read using an ELx808 automatic microplate reader (Bio-Tek Instruments) at 630 nm.

Viral entry assay (BLAM assay). The viral entry assay was performed as previously described (11). Briefly, viruses were generated by co-transfection of HEK293T cells with three plasmids: pNL4-3, pAdvantage (Promega) and pCMV4-3BlaM-Vpr (kindly provided by Dr. Warner C. Greene) (at a ratio of 6:1:2). Supernatant was harvested at 48 hours posttransfection, concentrated, and then used for infection as suggested (11). Flow cytometry was performed using a Becton Dickinson LSR II (Becton Dickinson). β-lactamase and CCF2 measurements were performed using a 407-nm violet laser with emission filters of 525/50 nm (green fluorescence) and 440/40 nm (blue fluorescence), respectively. Green and blue emission spectra were separated using a 505LP dichroic mirror. The UV laser was turned off during the analysis. Data analysis was performed using FlowJo software (FlowJo).

Viral attachment assay. Virion particles produced in the presence of PSGL-1 or the empty vector were incubated with HelaJC.53 cells (pre-chilled at 4°C for 1 hour) at 4°C for 2 hours. The cells were then washed extensively (5 times) with cold PBS buffer and then lysed with LDS lysis buffer (Invitrogen) for analysis by Western blot.

Infectivity assays. For flow cytometry-based infectivity assay, virus particles were produced in HEK293T cells by cotransfection with pNL4-3, pNL4-3ΔVpu, or pNL4-3ΔNef with pCMV3-PSGL-1, pCMVCD43, or pCMV3-Empty, or by cotransfection with pNL4-3/KFS, pHCMV-G, and pCMV3-PSGL-1 or pCMV3-Empty vector (using the indicated plasmid inputs) in a 6-well plate with Lipofectamine 2000 (Invitrogen). Viral particles were also produced in CEM-SS cells by electroporation. Briefly, CEM-SS cells (2 millions) were electroporated with pNL4-3 (2 μg) plus 400 ng of pCMV3-PSGL-1 or pCMV3-Empty using a T cell electroporation kit (Lonza). Viral particles were harvested at 3 days post electroporation. Rev-A3R5-GFP cells were infected with each of the indicated viruses (0.2-0.5 million cells/infection). The cells were then washed and cultured in fresh media. Flow cytometry analysis of GFP expression was performed on the indicated days. The percentage of GFP+ cells was quantified.

For luciferase-based, single-cycle infectivity assays, RT-normalized virus stocks were used to infect the $CD4^+/CXCR4^+/CCR5^+$ HeLa derivative TZM-bl. This indicator cell line contains integrated copies of the β-galactosidase and luciferase genes under the control of the HIV-1 LTR. Infection efficiency was determined by measuring luciferase activity 2 days postinfection. For infectivity assays in HeLa JC53-PSGL-1 and HeLa-JC53-empty cell lines, the cells were seeded in 6-well plates at a density of 0.2 x 10^6/well 24 hours prior to infection. Cells were infected with the indicated p24 equivalents of either WT NL4-3, NL43ΔVpu, or NL43ΔNef. Viral replication was quantified by virion p24 released into the medium by p24 ELISA. For MLV (murine leukemia virus) virion infectivity, MLV-GFP reporter virus was assembled by co-transfecting HEK293T cells (in 6-well plate) with pSV-Ψ-MLV-env^- (0.375 μg), pRetroQ-AcGFP1-N1 (0.5 μg), pHCMV-G (0.125 μg), and pCMV3-PSGL1 or an empty vector at the indicated dosages. An equal amount of DNA was used all transfections. Viral supernatants were harvested 48h post transfection and used to infect HEK293T cells for 6 hours in the presence of Infectin (Virongy, Manassas, VA). Cells were washed to remove virus and Infectin, and cultured for 48 hours for flowcytometry analyses.

To determine the effect of PSGL-1 on influenza A virus replication, HEK293T and MDCK cells were co-cultured at approximately 70% confluence in 6-well plates and transfected with either empty vector or pCMV3-PSGL-1 (both vectors at 1.0 or 3.0 μg), together with an eight-plasmid influenza A/WSN/33 reverse genetics system (RGS) (1.0 μg of each plasmid). Transfection reaction was prepared with PEI (polyethylenimine). Culture supernatants were collected at 16 and 24 h post-transfection and titrated in MDCK cells to determine end-point titers ($TCID_{50}$ per ml).

shRNA knockdown of PSGL-1. Lentiviral vectors carrying shRNAs against PSGL-1 or non-target control (NTC) (Sigma MISSION shRNA, PSGL-1 TRCN0000436811 or shRNA NTC) were purchased from Sigma. Virion particles were assembled by cotransfecting HEK293T cells with 0.5 µg of pHCMV-G, 1.5 µg pCMV-ΔR8.2, and 2 µg of lentiviral vectors using Lipofectamine 3000 (Invitrogen). Supernatant was collected at 48 hours post co-transfection, and then filtered through 0.45um filter. Virion particles were used to transduce Jurkat T cells for 6 hours. Cells were then washed twice and cultured in fresh media for 3 days, and then selected in puromycin (4 µg /ml) for one to two weeks. PSGL-1 knockdown was confirmed by surface staining with an anti-PSGL-1 antibody (KPL-1) (BD Pharmingen) followed by staining with Alexa Fluor 488-conjugated goat anti-mouse secondary antibody (Invitrogen). PSGPL-1 knockdown or NTC control cells (2 x 10^6) were also transfected with 2 µg of HIV-1(NL4-3) DNA by electroporation using a T cell electroporation kit (Lonza). Viruses were harvested and used for the infection of Rev-A3R5-GFP cells (20 ng p24 per infection). Flow cytometry analysis of GFP expression was performed on the indicated days. Lentiviral vector-mediated ShRNA knockdown of PSGL-1 in primary CD4 T cells was performed as described previously (1). Briefly, blood resting CD4 T cells were purified by negative depletion, transiently stimulated with anti-CD3/CD28 beads (1 -2 beads per cell) for 12 hours, and then transduced with the lentiviral vectors carrying shRNAs against PSGL-1 or non-target control (NTC) (Sigma MISSION shRNA, PSGL-1 TRCN0000436811 or shRNA NTC). Following transduction, the beads were removed at 12 hours, and cells were cultured for 3 days, and then analyzed from surface PSGL-1 expression. Cells were also subsequently transfected with HIV-1(NL4-3) DNA by electroporation using a T cell electroporation kit (Lonza). HIV-1 viral replication was monitored by harvesting cell culture supernatant, and HIV p24 was detected by an in-house p24 ELISA kit.

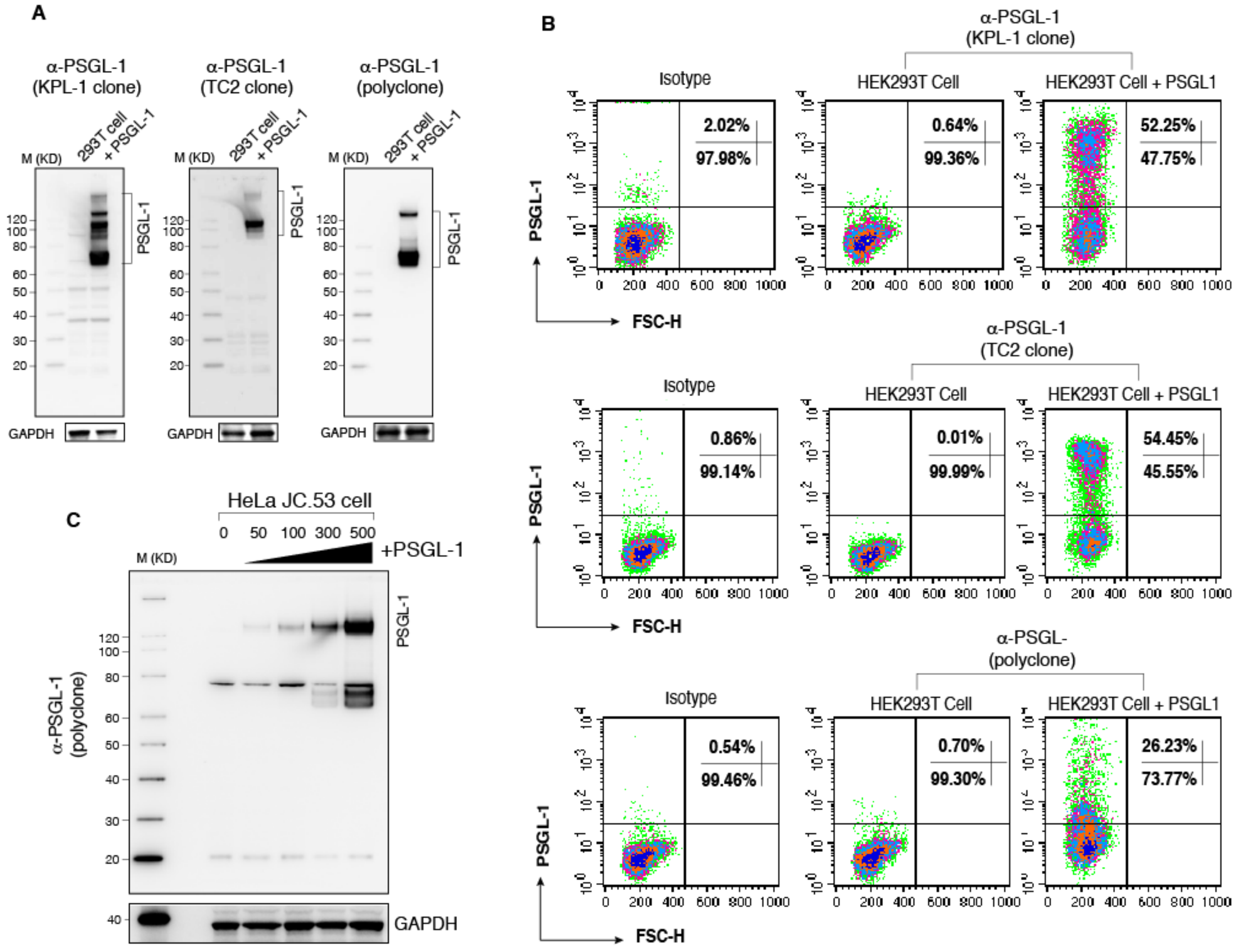

Fig. S1. Validation of PSGL-1 expression following transfection of HEK293T and HeLa JC.53 cells. **A** and **B)** HEK293T cells were transfected with a PSGL-1 expression vector (pCMV3-PSGL-1), and analyzed by western blot using 3 different commercial antibodies (**A**). Expression of PSGL-1 on the surface was analyzed by surface staining and flow cytometry. Shown are the percentages of cells with high or low PSGL-1 staining in each panel. **C)** HeLa JC.53 cells were transfected with pCMV3-PSGL-1 at the indicated inputs (ng). PSGL-1 expression was analyzed by western blot at 48 hours posttransfection using anti-PSGL-1 polyclonal antibodies. GAPDH was similarly analyzed as a loading control.

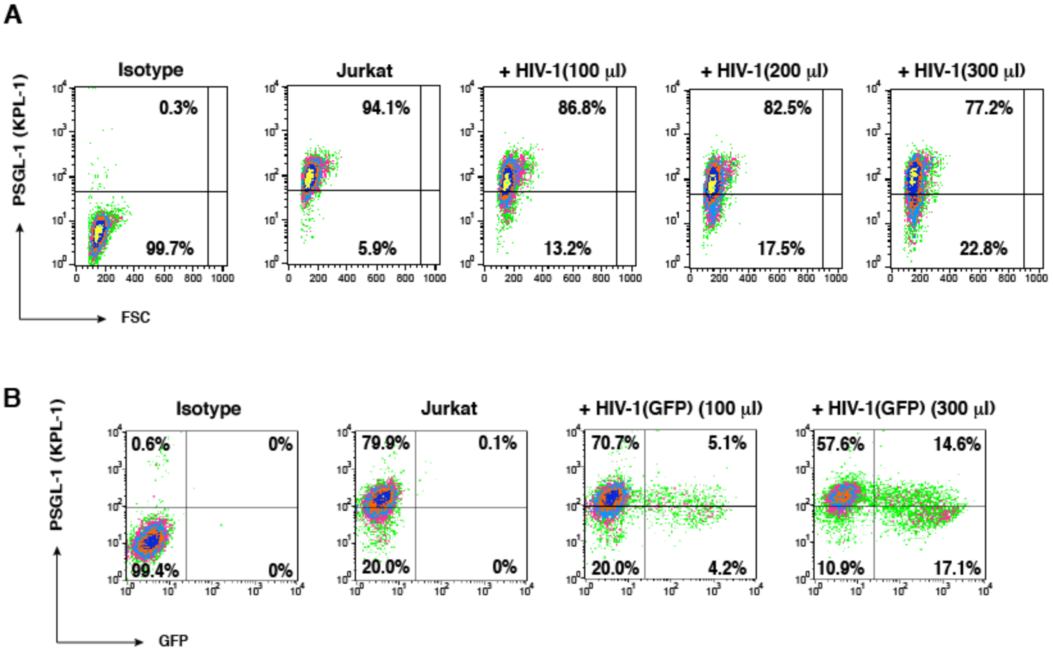

Fig. S2. HIV-1 dose-dependent downregulation of PSGL-1 in Jurkat T cells. **A**) Jurkat T cells were infected with different inputs of HIV-1, washed and cultured for 3 days, and then stained for surface PSGL-1 expression, and analyzed by flow cytometry. Shown are the percentages of cells with high or low PSGL-1 staining in each panel. **B**) Cells were similarly infected with different inputs of a HIV-1 reporter virus, HIV-1(GFP), and then stained for surface PSGL-1 expression. Shown are the percentages of the GFP+ or GFP- cells with low or high PSGL-1 staining in each panel.

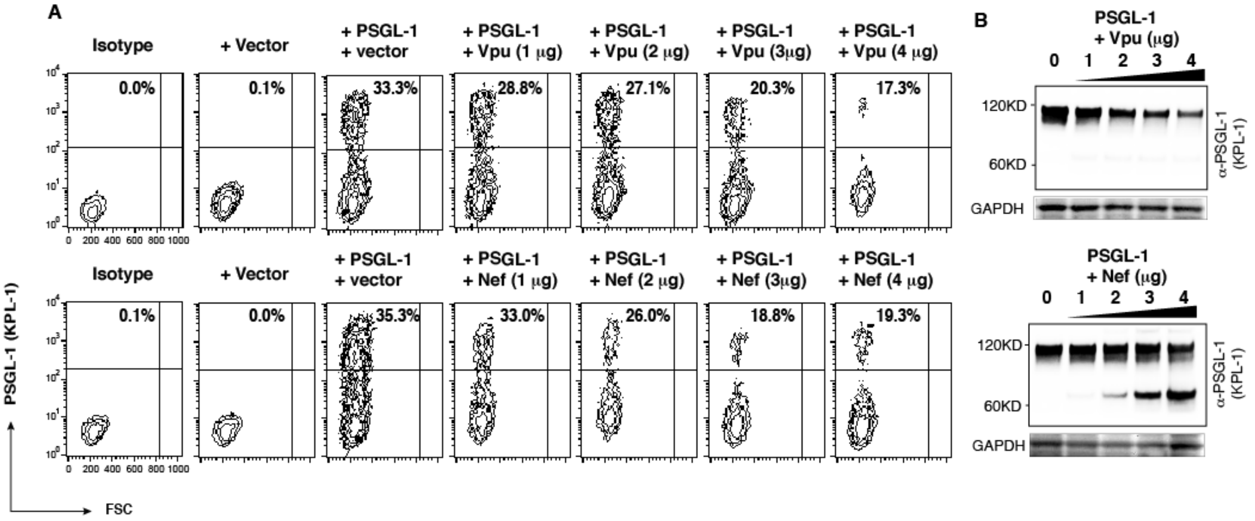

Fig. S3. Downregulation of PSGL-1 from the cell surface by Vpu and Nef. A) HEK293T cells were cotransfected with PSGL-1 (100 ng) and a Vpu or Nef expression vector at various DNA inputs. Surface PSGL-1 expression was quantified and shown as the percentages of cells expressing PSGL-1. For controls, an empty vector was used (+Vector). The same amount of DNA was used in all transfections. **B)** Levels of intracellular PSGL-1 in Vpu- or Nef-cotransfected cells were quantified by western blot at 48 hours post-cotransfection.

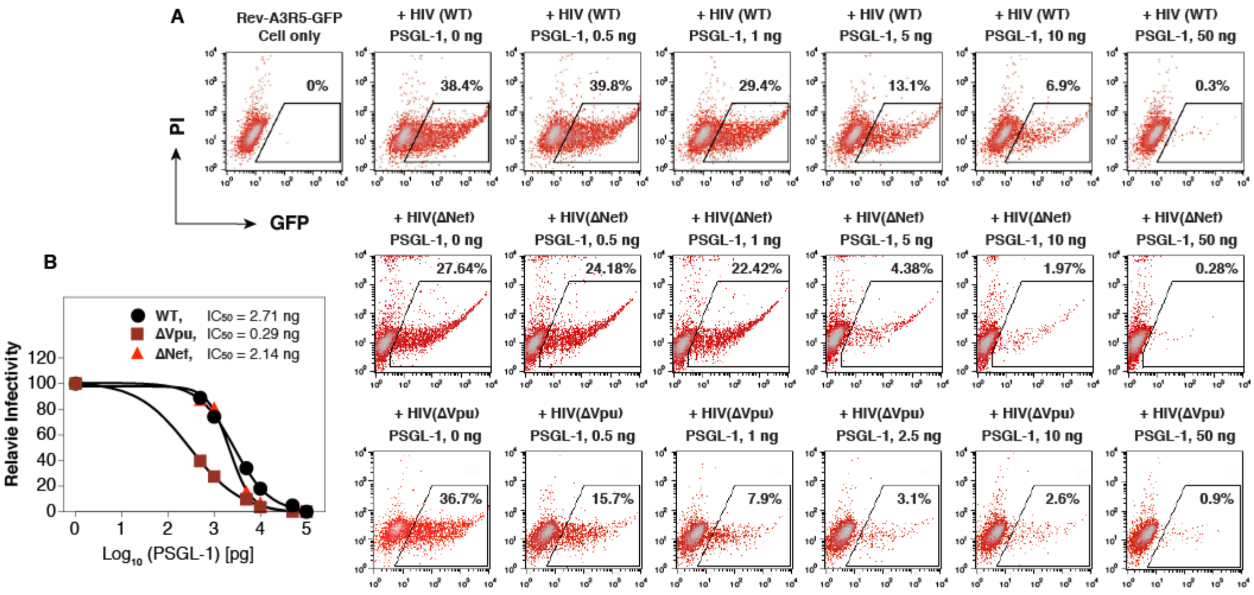

Fig. S4. **Comparison of Vpu and Nef in antagonizing PSGL-1**. **A**) HEK293T cells were cotransfected with various amounts of PSGL-1 DNA (0.5 – 50 ng) plus 1 µg HIV(NL4-3) WT, HIV(ΔVpu), or HIVΔNef DNA. Virions were harvested and normalized for p24. Viral infectivity was quantified by infecting the T-cell line-derived Rev-A3R5-GFP indicator cell line. HIV-1 replication was quantified by GFP expression. Shown are the percentages of GFP+ cells at 48-72 hours postinfection. **B**) The PSGL-1 dose-dependent inhibition curve was plotted.

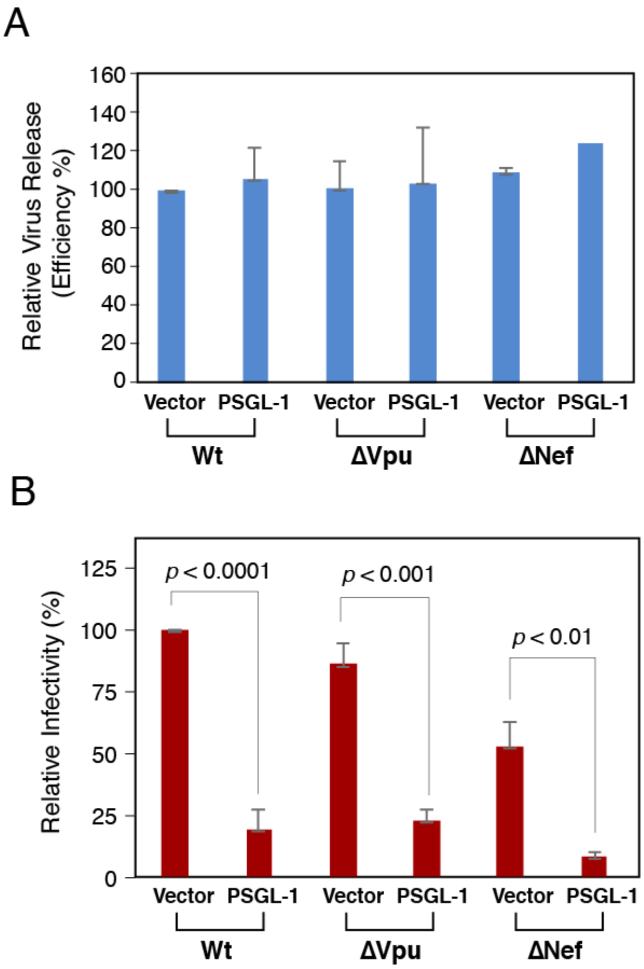

Fig. S5. PSGL-1 inactivates HIV-1 infectivity. **A)** HEK293T cells were cotransfected with 1 µg of HIV-1(NL4-3), HIV-1(ΔVpu), or HIV-1(ΔNef) DNA plus 100 ng PSGL-1 expression vector. One day posttransfection, virus supernatants were harvested, and virus release efficiency (VRE) was quantified by western blot as the amount of virion-associated p24 (CA) relative to total Gag in cell and virus lysates. VRE was set to 100% for WT HIV-1 in the absence of PSGL-1. **B)**, At 30 hours posttransfection, virus supernatants were harvested, and an aliquot was used for reverse transcriptase (RT) and infectivity assays. RT-normalized virus was used to infect TZM-bl cells for 2 days, and luciferase activity was measured. Infectivity of WT HIV-1 in the absence of PSGL-1 was set to 100%. Data shown are ± SD from three independent experiments. Data were evaluated for statistical significance using the unpaired *t* test.

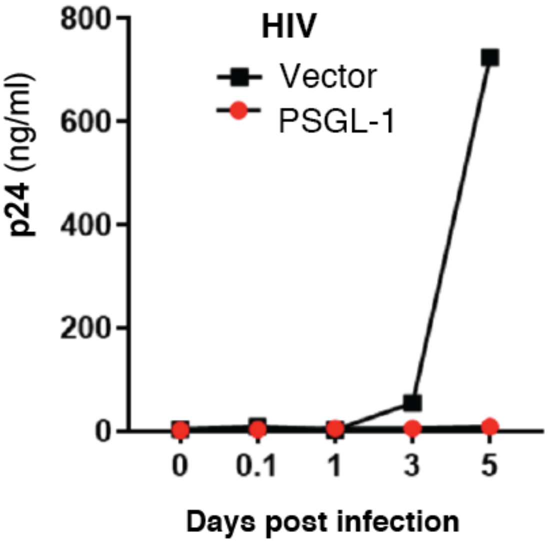

Fig. S6. PSGL-1 blocks the establishment of a spreading HIV-1 infection. HEK293T cells (3 x 10^6) were cotransfected with 12 µg of HIV(NL4-3) plus 2.4 µg pCMV-PSGL-1 or an empty vector. Viruses were harvested at 48 hours post-transfection and used to infect A3R5.7 CD4 T cells. After infection for 4 hours, cells were washed and cultured for 5 days. HIV replication was analyzed by p24 release.

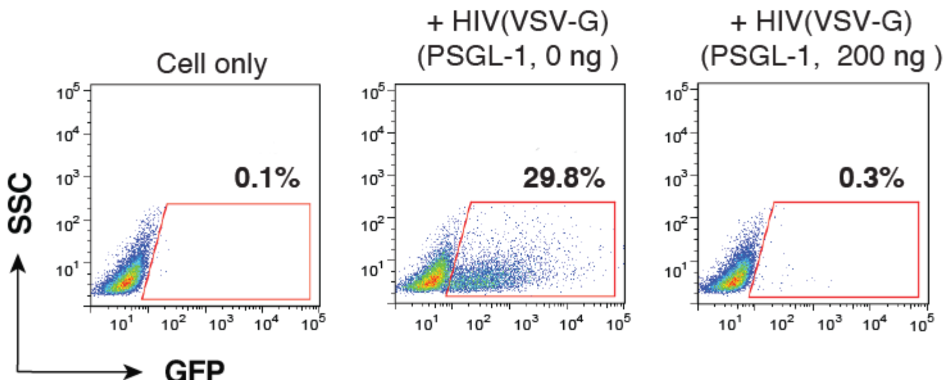

Fig. S7. PSGL-1 inactivates the infectivity of VSV-G-pseudotyped HIV-1 virions. HEK293T cells (2 x 10^5) were cotransfected with 1 µg of the Env(-) clone pNL4-3/KFS, 1 µg pHCMV-G, and 200 ng pCMV-PSGL-1 or an empty vector. Virus supernatants were harvested at 48 hours posttransfection, and used to infect Rev-A3R5-GFP cells. After infection for 4 hours, cells were washed and cultured in medium for 72 hours. The percentages of infected (GFP+) cells were quantified by flow cytometry.

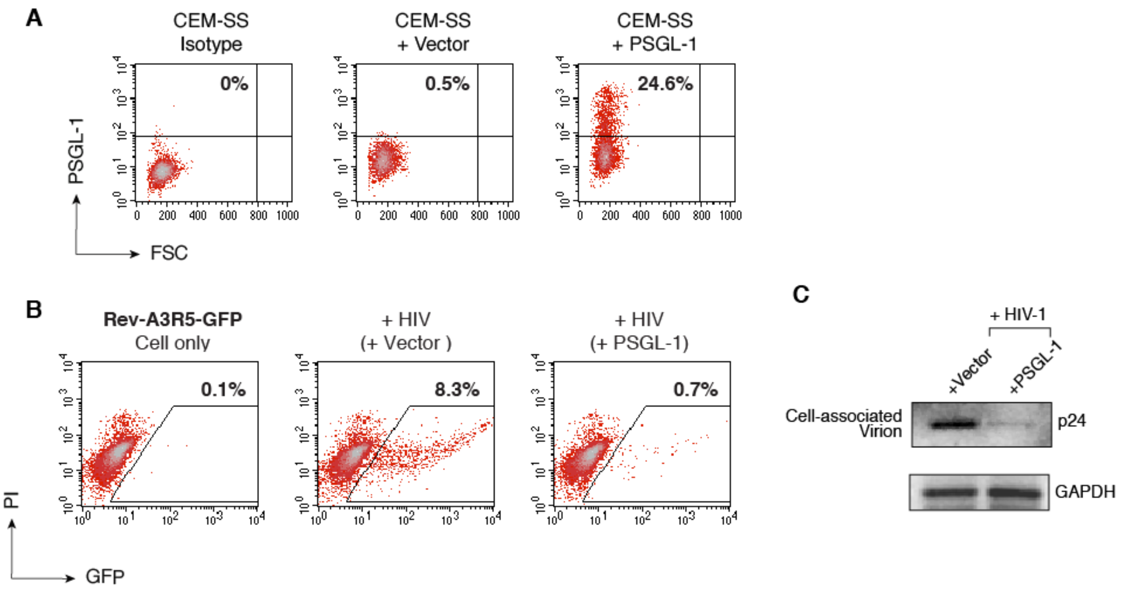

Fig. S8. PSGL-1 inactivates the infectivity of HIV-1 virions produced from CEM-SS cells. A) CEM-SS cells were electroporated with HIV-1(NL4-3) DNA plus PSGL-1 DNA or an empty vector. PSGL-1 surface expression was quantified at 3 days post-electroporation. **B**) To quantify HIV infectivity, virions were harvested at 3 days post-electroporation, and used to infect Rev-A3R5-GFP cells, using an equal amount of p24 for infection. GFP expression was quantified at 3 days post-infection. **C**) Virions produced in the presence of PSGL-1 or the empty vector were assayed for attachment to target HeLa JC.53 cells at 4°C for 2 hours. Cells were washed and then analyzed by western blot for bound p24.

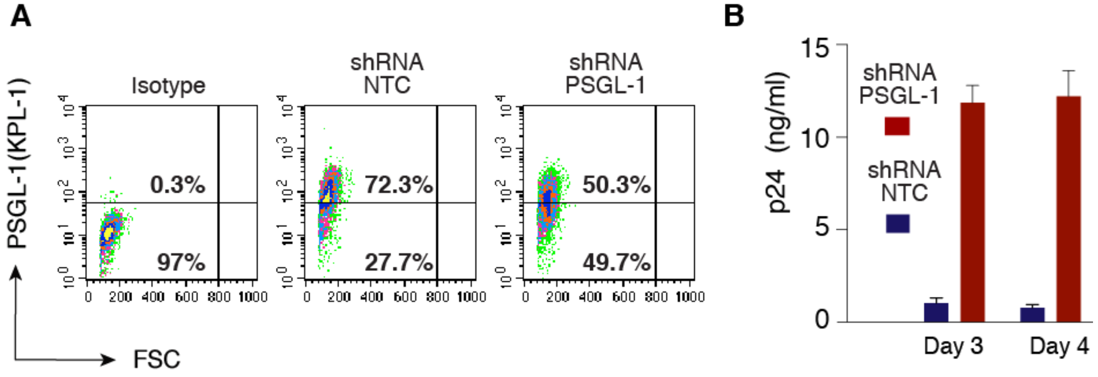

Fig. S9. shRNA knockdown of PSGL-1 in primary CD4 T cells enhances HIV-1 replication. **A**) Blood resting CD4 T cells were purified by negative depletion, activated with anti-CD3 and CD28 magnetic beads, and then transduced with a lentiviral vector expressing shRNA against PSGL-1 (shRNA PSGL-1) or a non-target sequence (shRNA NTC). PSGL-1 surface expression was quantified at day 3 post-transduction. **B**) Cells were also electroporated with HIV-1(NL4-3) DNA, and viral replication in the PSGL-1 knockdown or the control NTC CD4 T cells was quantified by measuring p24 in the supernatant.

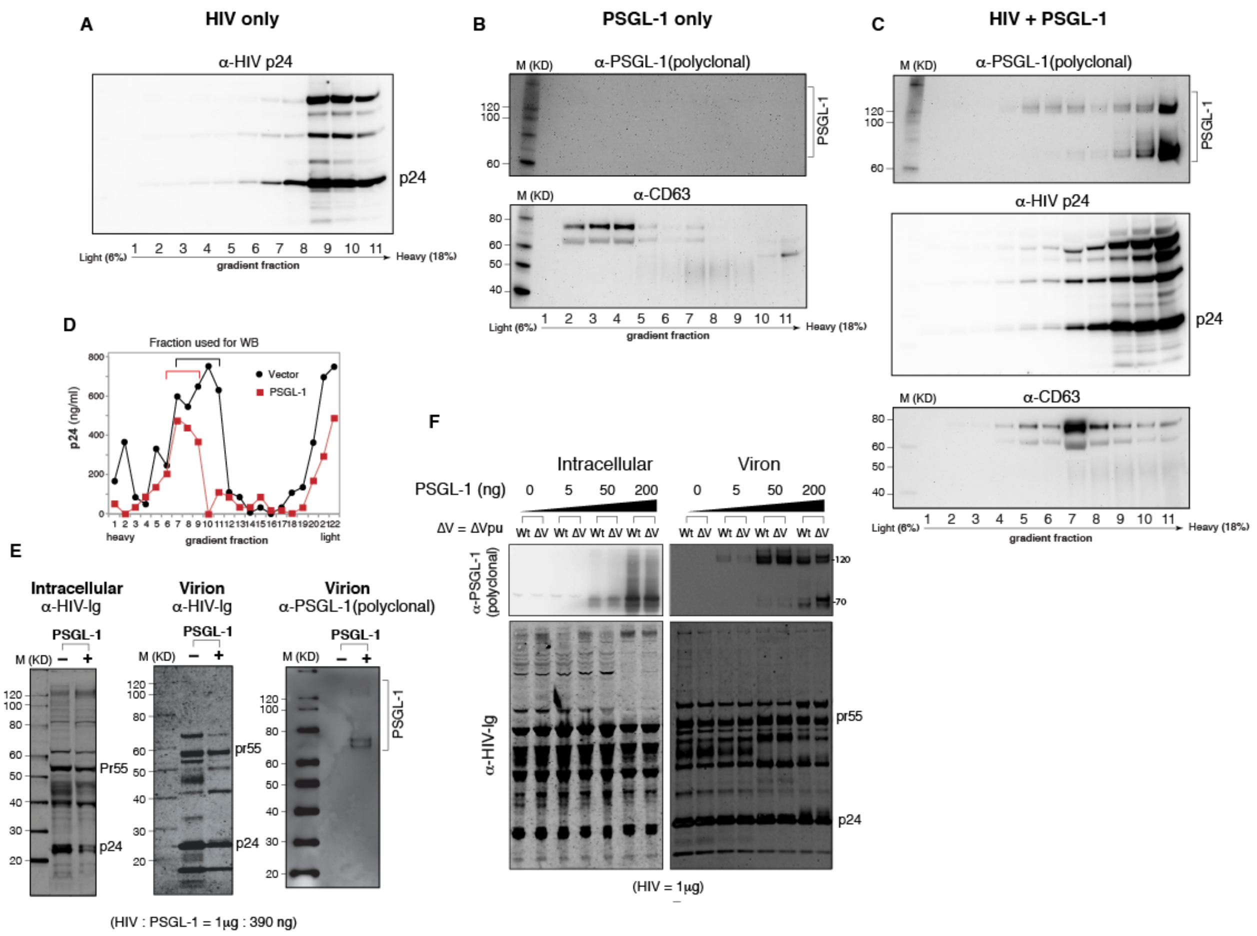

Fig. S10. Virion incorporation of WT PSGL-1. A to **C)** HEK293T cells were transfected with HIV-1(NL4-3) (1 μg , HIV only), or transfected with PSGL-1 (200 ng, PSGL-1 only), or cotransfected with 1 μg HIV-1(NL4-3) plus 200 ng of PSGL-1 DNA (HIV + PSGL-1). Supernatants were harvested at 48 hours, filtered, concentrated, and purified by ultra-speed centrifugation through a 6%-18% OptiPrep gradient. PSGL-1 and viral p24 proteins in each fraction were analyzed by western blot using antibodies against PSGL-1 (polyclonal) or HIV-1 p24 (anti-p24). For comparison, an anti-CD63 antibody was used to identify the fractions also containing exosomes. **D** and **E**) HEK293T cells were cotransfected with HIV-1(NL4-3) DNA plus PSGL-1 DNA at the indicated ratios. Virus particles were harvested at 48 hours and purified by ultra-speed centrifugation through an OptiPrep gradient (6%-18%). The presence of viral p24 in each fraction was directly analyzed by p24 ELISA (**D**). Expression of viral proteins in cotransfected cells was analyzed by western blot using antibodies against HIV-1 proteins (anti-HIV serum); virion proteins were also analyzed by harvesting and pelleting the peak virion fraction, and performing western blot using antibodies against PSGL-1 (polyclonal) or HIV-1 proteins (anti-HIV serum). Positions of Gag precursor protein (pr55) and p24 (CA) are indicated (**E**). **F)** HEK293T cells were cotransfected with varying amounts of PSGL-1 DNA plus 1 μg HIV-1(NL4-3) or HIV(ΔVpu) DNA at the indicated ratios. Virus particles were harvested at 48 hours and purified by ultra-speed centrifugation through an OptiPrep gradient (6%-18%). Intracellular and virion proteins were similarly analyzed by western blots for PSGL-1 and HIV-1 proteins.

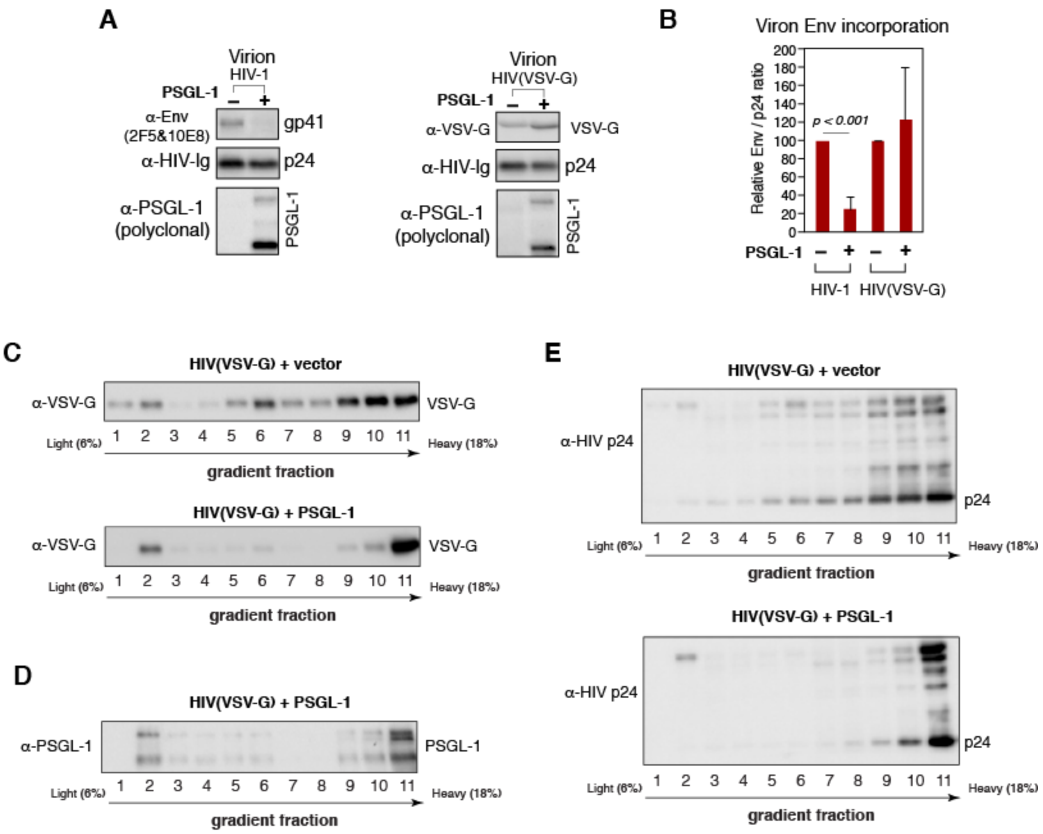

Fig. S11. PSGL-1 disrupts HIV-1 Env but not VSV-G incorporation into virions. **A** and **B**) HEK293T cells were cotransfected with HIV-1 in the presence of PSGL-1 or an empty vector. Cells were also cotransfected with pNL4-3/KFS plus pCMV-VSV-G in the presence of PSGL-1 or an empty vector. Virus particles produced were analyzed by western blot using antibodies against PSGL-1 (polyclonal), HIV proteins (anti-HIV serum), HIV-1 Env proteins (2F5 and 10E8), or VSV-G. **C** to **E)** Effects of PSGL-1 on virion incorporation of VSV-G. HEK293T cells were cotransfected with 10 µg pNL4-3/KFS, 2 µg pCMV-VSV-G, and 2 µg PSGL-1 or a control empty vector. Viral particles were harvested at 48 hours, filtered, concentrated, and purified by ultra-speed centrifugation through a 6%-18% OptiPrep gradient. Virion proteins were analyzed by pelleting each fraction, and performing western blot using antibodies against VSV-G (**C**), PSGL-1 (**D**), or HIV-1 p24 (anti-HIV p24) (**E**).

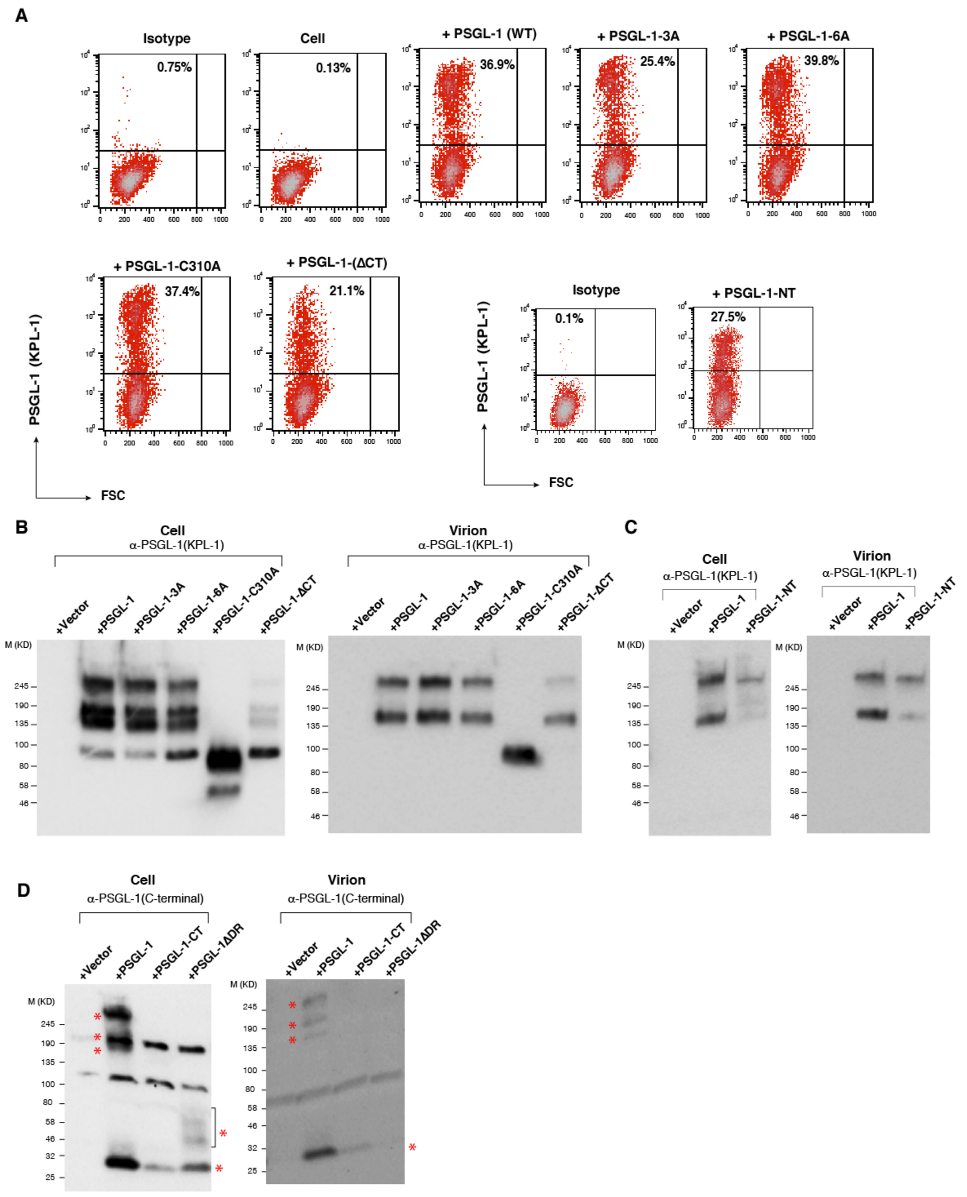

Fig. S12. Expression and virion incorporation of PSGL-1 mutants. A) HEK293T cells were transfected with PSGL-1 DNA (500 ng) or each of the PSGL-1 mutant DNA (500 ng). Expression of PSGL-1 was quantified by surface staining with an anti-PSGL-1 antibody (KPL-1 clone) at 48 hours. **B** to **C**). HEK293T cells were cotransfected with HIV-1 DNA (1 μg) plus PSGL-1 DNA or each of the PSGL-1 mutant DNAs (250 ng of PSGL-1, PSGL-1-3A, PSGL-1-6A and PSGL-1-C310A mutants; 500 ng of PSGL-1-ΔCT, pRetroPSGL-1, PSGL-1-NT, PSGL-1-CT and PSGL-1ΔDR). Expression of PSGL-1 or PSGL-1 mutants in cotransfected cells was detected by western blot. Viral particles were harvested, and virion incorporation of PSGL-1 was detected by western blotting using the anti-PSGL-1 antibody KPL-1. **D**) For the N-terminal truncation mutants, PSGL-1-CT and PSGL-1ΔDR, cellular expression and virion incorporation were detected by western blotting using the anti-PSGL-1 antibody ab66882 (Abcam). The expected protein sizes of PSGL-1 and the mutant proteins are labeled with red asterisk.

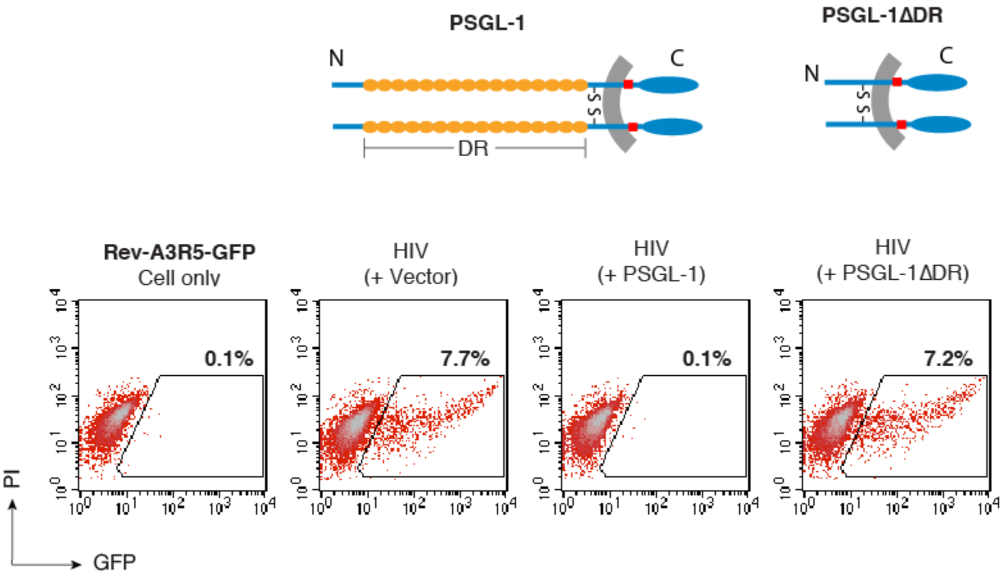

Fig. S13. The extracellular, N-terminal DR domain of PSGL-1 is required to block HIV-1 infectivity. HEK293T cells were cotransfected with HIV(NL4-3) DNA (1 µg) plus vectors expressing PSGL-1 or PSGL-1 decameric repeat (DR) truncation mutant PSGL-1ΔDR (400 ng). Virions were harvested at 48 hours post-transfection and normalized for p24, and viral infectivity was quantified by infecting Rev-A3R5-GFP indicator cells. HIV-1 replication was quantified by GFP expression. An empty vector was used as the cotransfection control (+Vector).

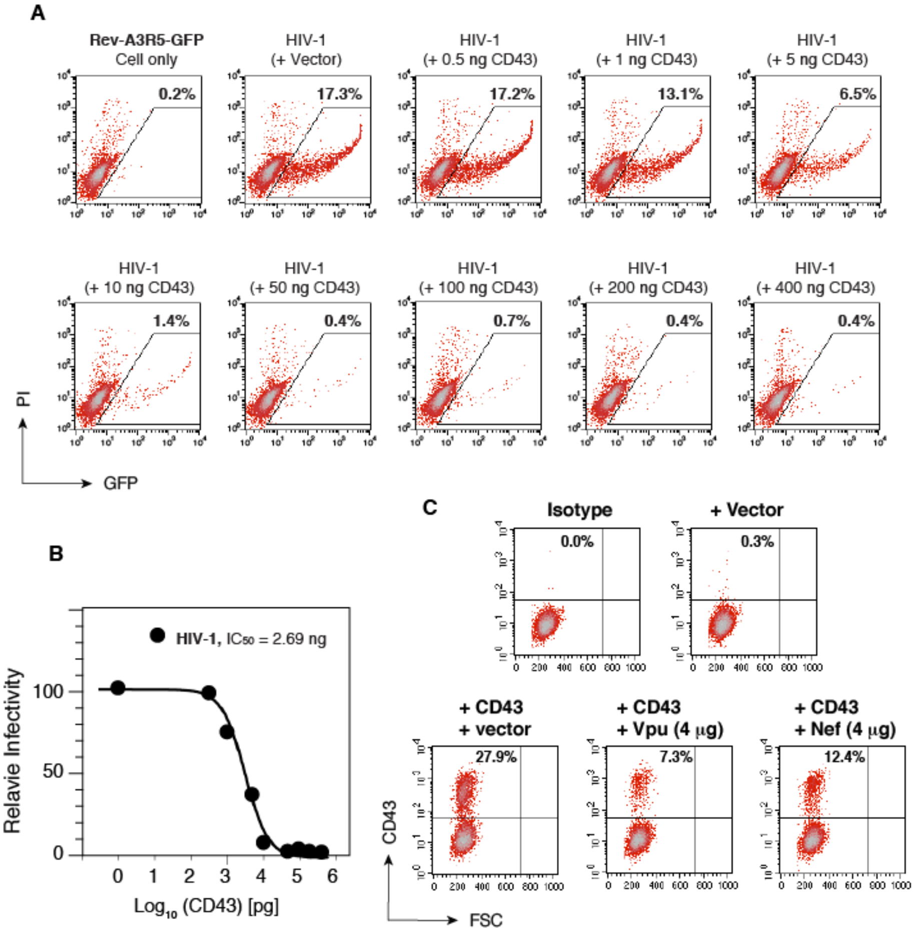

Fig. S14. CD43 inactivates HIV-1 virion infectivity. A) HEK293T cells were cotransfected with various amounts of CD43 DNA (0.5 – 400 ng) plus 1 µg HIV(NL4-3) DNA. Virions were harvested at 48 hours and normalized for p24. Viral infectivity was quantified by infecting Rev-A3R5-GFP indicator cells. HIV-1 replication was quantified by GFP expression. Shown are the percentages of GFP+ cells at 48 hours postinfection. **B**) The CD43 dose-dependent inhibition curve was plotted. **C**) Downregulation of CD43 from the cell surface by Vpu and Nef. HEK293T cells were cotransfected with CD43 (100 ng) and a Vpu or Nef expression vector (4 µg). Surface CD43 expression was quantified and shown as the percentage of cells expressing CD43. For controls, an empty vector was used (+vector). The same amount of DNA was used in all transfections.

References

1. Yoder A*, et al.* (2008) HIV envelope-CXCR4 signaling activates cofilin to overcome cortical actin restriction in resting CD4 T cells. *Cell* 134(5):782-792.
2. Wei X*, et al.* (2002) Emergence of resistant human immunodeficiency virus type 1 in patients receiving fusion inhibitor (T-20) monotherapy. *Antimicrob Agents Chemother* 46(6):1896-1905.
3. Grover JR, Veatch SL, & Ono A (2015) Basic motifs target PSGL-1, CD43, and CD44 to plasma membrane sites where HIV-1 assembles. *J Virol* 89(1):454-467.
4. Llewellyn GN, Grover JR, Olety B, & Ono A (2013) HIV-1 Gag associates with specific uropod-directed microdomains in a manner dependent on its MA highly basic region. *J Virol* 87(11):6441-6454.
5. Tauxe C*, et al.* (2008) P-selectin glycoprotein ligand-1 decameric repeats regulate selectin-dependent rolling under flow conditions. *J Biol Chem* 283(42):28536-28545.
6. Yee JK*, et al.* (1994) A general method for the generation of high-titer, pantropic retroviral vectors: highly efficient infection of primary hepatocytes. *Proc Natl Acad Sci U S A* 91(20):9564-9568.
7. Freed EO, Delwart EL, Buchschacher GL, Jr., & Panganiban AT (1992) A mutation in the human immunodeficiency virus type 1 transmembrane glycoprotein gp41 dominantly interferes with fusion and infectivity. *Proc Natl Acad Sci U S A* 89(1):70-74.
8. Levy DN, Aldrovandi GM, Kutsch O, & Shaw GM (2004) Dynamics of HIV-1 recombination in its natural target cells. *Proc Natl Acad Sci U S A* 101(12):4204-4209.
9. Trinite B*, et al.* (2014) Suppression of Foxo1 activity and down-modulation of CD62L (L-selectin) in HIV-1 infected resting CD4 T cells. *PLoS One* 9(10):e110719.
10. Trinite B, Chan CN, Lee CS, & Levy DN (2015) HIV-1 Vpr- and Reverse Transcription-Induced Apoptosis in Resting Peripheral Blood CD4 T Cells and Protection by Common Gamma-Chain Cytokines. *J Virol* 90(2):904-916.
11. Cavrois M, De Noronha C, & Greene WC (2002) A sensitive and specific enzyme-based assay detecting HIV-1 virion fusion in primary T lymphocytes. *Nat Biotechnol* 20(11):1151-1154.
